# A data driven approach reveals disease similarity on a molecular level

**DOI:** 10.1038/s41540-019-0117-0

**Published:** 2019-10-25

**Authors:** Kleanthi Lakiotaki, George Georgakopoulos, Elias Castanas, Oluf Dimitri Røe, Giorgos Borboudakis, Ioannis Tsamardinos

**Affiliations:** 1 Computer Science Department, University of Crete, Heraklion, Greece; 2 Laboratory of Experimental Endocrinology, School of Medicine, University of Crete, Heraklion, Greece; 3 Norwegian University of Science and Technology, Department of Clinical Research and Molecular Medicine, Trondheim, Norway; 4 Levanger Hospital, Nord-Trøndelag Hospital Trust, Cancer Clinic, Norway; 5 Clinical Cancer Research Center, Department of Clinical Medicine, Aalborg, Denmark; 6 Gnosis Data Analysis PC, Heraklion Crete, Greece; 7 Institute of Computational and Applied Mathematics, Foundation for Research and Technology, Heraklion, Greece

**Keywords:** Information theory, Computer science

## Abstract

Could there be unexpected similarities between different studies, diseases, or treatments, on a molecular level due to common biological mechanisms involved? To answer this question, we develop a method for computing similarities between empirical, statistical distributions of high-dimensional, low-sample datasets, and apply it on hundreds of -omics studies. The similarities lead to dataset-to-dataset networks visualizing the landscape of a large portion of biological data. Potentially interesting similarities connecting studies of different diseases are assembled in a disease-to-disease network. Exploring it, we discover numerous non-trivial connections between Alzheimer’s disease and schizophrenia, asthma and psoriasis, or liver cancer and obesity, to name a few. We then present a method that identifies the molecular quantities and pathways that contribute the most to the identified similarities and could point to novel drug targets or provide biological insights. The proposed method acts as a “statistical telescope” providing a global view of the constellation of biological data; readers can peek through it at: http://datascope.csd.uoc.gr:25000/.

## Statistics reveal molecular sources of disease similarities

Different phenotypes (e.g., diseases) may be distinct on a macroscopic level, however, they may share several common biological mechanisms on a molecular level. The molecular similarities should be reflected as statistical similarities in corresponding -omics data measurements. A method is presented to identify these statistical similarities. It is then applied to hundreds of public -omics datasets stemming from different studies, creating a network of diseases (phenotypes in general) whose statistical molecular patterns are similar. We discover numerous non-trivial connections on a molecular level between Alzheimer's disease and schizophrenia, asthma and psoriasis, liver cancer and obesity, to name a few. The quantities and pathways that contribute the most to the identified similarities are also reported. They could point to novel drug targets or biological insights.

## Introduction

Public biological data repositories currently hold tens of thousands of (*bio*)-*datasets*. For example, as of October 2019, the NCBI Gene Expression Omnibus (GEO)^1^ contains 3,263,365 microarray and RNA-Seq profiles, grouped into 119,386 data series. Each dataset studies a specific biological question, regarding a disease, a treatment, or a phenotype. Examples include finding the gene expression differences between malignant and benign breast tissue or creating a diagnostic model between primary and metastatic lung cancer tumors. Data analysis methods then typically focus on individually analyzing each dataset, like a “statistical microscope”. However, the question arises: how do the measurements from these studies compare against each other, what are their relations, and what is the collective, emerging picture and biological intuition they provide? Can we construct and look through a “statistical telescope” instead? Could it be that different diseases, treatments, other experimental or sampling conditions induce similar biological molecular patterns pointing to common pathophysiological pathways? Their identification could accelerate the deeper understanding of human pathology and the exploitation of clinical study results.

**Fig. 1:**
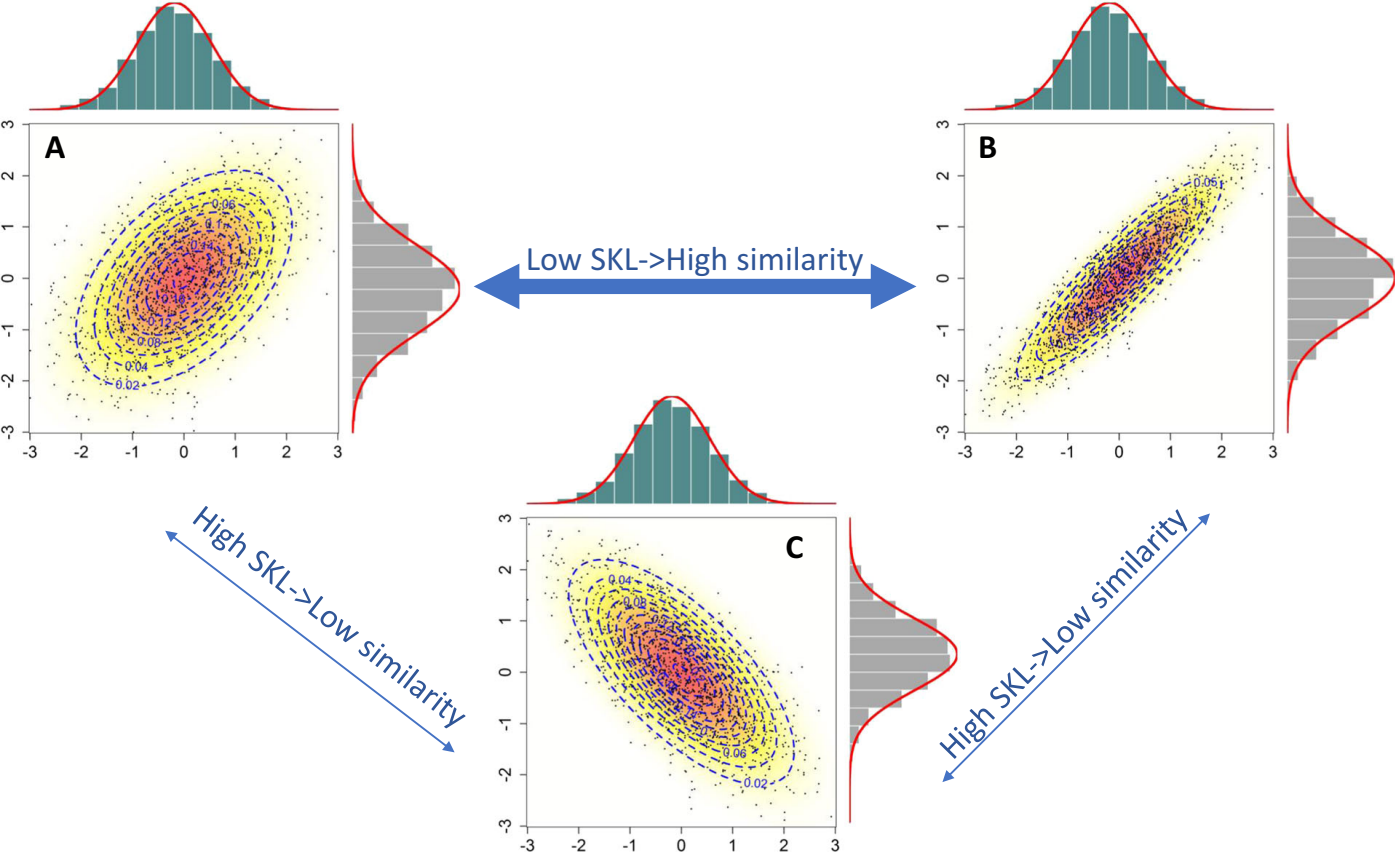
Comparing empirical, high-dimensional statistical distributions. A visual example of pairwise comparison of three, standardized, bivariate normal distributions. Each point corresponds to a molecular profile measuring just two quantities. Contours are drawn to indicate regions of equal probability density. The comparison is generally based on the covariance matrix. In this case, what matters is the single covariance between the two quantities measured: positive for datasets *A* and *B*, and negative for *C*. Distribution of *A* is more similar to *B* than to *C*. In high-dimensional spaces, the contours become surfaces that form ellipsoids. Geometrically, the distributions are compared based on size and orientation of these ellipsoids. The metric of (dis)similarity proposed approximates the Symmetric Kullback–Leibler divergence and is denoted as *c-SKL*

To address these questions, we compute similarities between datasets, as distances between two empirical, multivariable, statistical distributions (see Fig. 1).

**Fig. 2:**
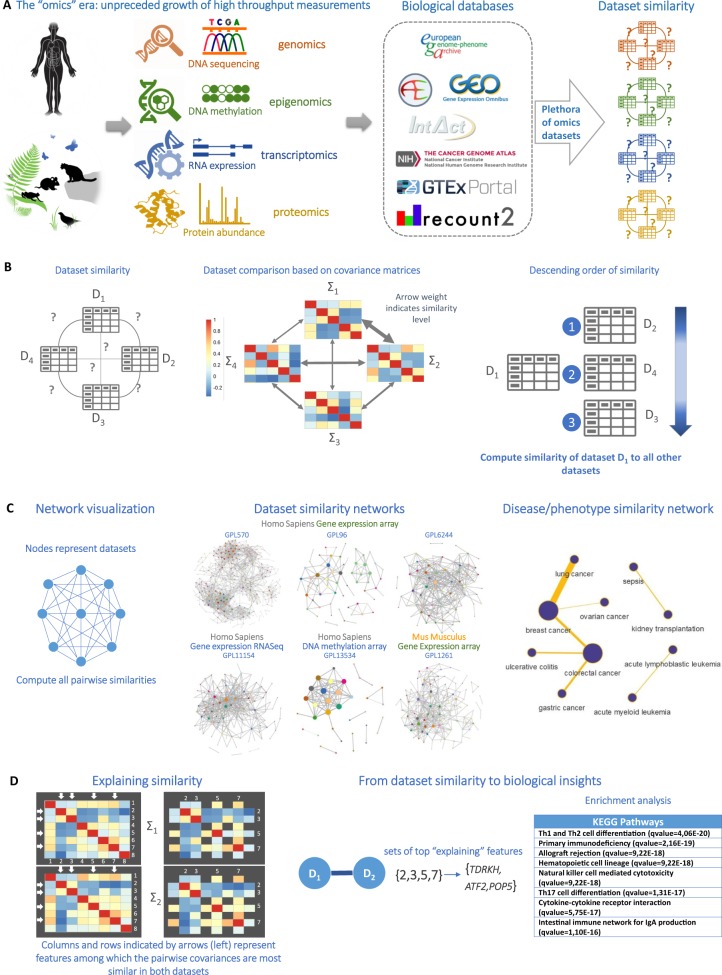
Towards a landscape of the biological dataome. **a** Problem definition: identify statistical similarities on a molecular level among public -omics datasets. **b** Compute all pairwise similarities based on the curated Symmetric Kullback–Leibler (*c-SKL*) divergence and the similarity of the covariance matrices. **c** The network of similarities among datasets of the same platform is visualized and explored for novel biological findings. The dataset similarity networks lead to a disease similarity network. **d** To gain intuition on the molecular underpinnings of interesting similarities, the molecular quantities that influence the c-SKL metric the most, are reported; these correspond to the same rows and columns in the covariance matrices not grayed out in the matrices on the right. They are used further as input for (pathway, gene ontology) enrichment analysis

A distributional similarity, referred simply as “similarity” hereafter, implies intuitively that molecular quantities measured in the two datasets are inter-correlated in a similar way, i.e., the covariance matrices are similar. We propose a generally applicable method, able to robustly estimate distributional distances, even when the dimensionality ranges into hundreds of thousands of observed molecular quantities (variables, features, attributes) and sample sizes as low as 40. It is emphasized that we compute the similarity of two datasets based only upon the statistical properties of the molecular measurements and ignoring any textual information. Similarities may arise due to several factors, such as concerning the same disease or treatment, measuring the same type of tissue, or employing sample analysis protocols and equipment that induce similar systematic batch effects; similarities may even arise between studies that share the same molecular profiles, a phenomenon that is surprisingly prevalent^2^ (networks depicting an alarming number of biological studies sharing molecular profiles can be visualized interactively at http://dataome.mensxmachina.org/networks). To account for shared profiles, in this work, we remove all datasets with at least one shared profile. However, when the method was applied, unexpected similarities between studies of different tissue or related to different pathologies were discovered, possibly attributed to a common etiology in their underlying biology. To enable expert inspection, we arranged the similarities into dataset-to-dataset networks, one for each measuring platform, where edges connect statistically significantly similar studies. The networks visualize the landscape of a sizable portion of the biological dataome and can be interactively explored online. To focus attention on the potentially most interesting similarities, we assemble the ones relating studies pertaining to different pathologies or phenotypes into a new type of network: a disease-to-disease network. Once an interesting dataset similarity is identified, it is natural to inquire the reason why. A second method is proposed to identify the molecular quantities (e.g., gene expressions) and corresponding enriched pathways, which contribute the most to the identified similarity, thus providing biological intuition regarding the common biological mechanisms involved. The overall approach is depicted in Fig. 2.

## Results

### Visualizing the landscape of large portions of the Biological Dataome

We used all the datasets from six measurement platforms that comply with certain criteria for sample size; datasets that share profiles with other datasets have been removed from consideration (SM1.1). Datasets include both micro-array technologies on transcriptomes and DNA methylomes, as well as RNA-seq technology; they also include measurements on subject tissues and cell lines. Specifically, the following platforms were included: (1) Homo Sapiens, gene expression, Affymetrix Human Genome U133 Plus 2.0 Array - (GPL570), (2) Homo Sapiens, gene expression, Affymetrix Human Genome U133A Array - (GPL96), (3) Homo Sapiens, gene expression, Affymetrix Human Gene 1.0 ST Array - (GPL6244), (4) Homo Sapiens, gene expression, Illumina HiSeq 2000 - (GPL11154), Homo Sapiens, DNA methylation, Illumina HumanMethylation450 BeadChip - (GPL13534), and Mus Musculus, gene expression, Affymetrix Mouse Genome 430 2.0 Array - (GPL1261). Overall 103,088 Homo Sapiens and Mus Musculus samples were employed in the subsequent analyses and results, grouped in 978 datasets and spanning more than 500 different diseases and phenotypes, as revealed by automated text analysis.^2^ Their descriptive presentation is shown in Supplementary Figs 1 and 2.

**Fig. 3:**
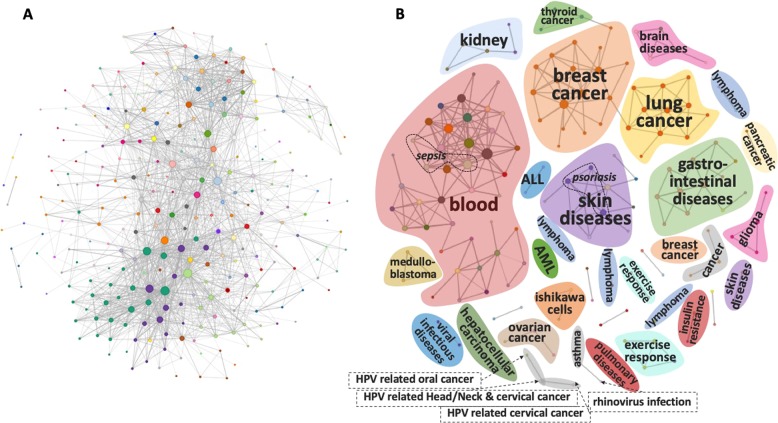
Dataset-to-dataset network of Homo Sapiens datasets measured by GPL570. **a** A telescopic view of all datasets measured by GPL570 (excluding studies with shared molecular profiles) including all of 1562 statistically significant edges. **b** The top 300 most statistically significant edges of the same network. Manual inspection and annotation, corresponding to the colored areas, show that nodes within communities (densely interconnected network regions) typically pertain to the same disease or tissue, which could explain the identified molecular similarities and provides biological evidence of the efficacy of the method

For each pair of datasets within the same platform, we computed the *c-SKL* as described in methods. The results are visualized as networks (graphs), where each node corresponds to a dataset (study) and each edge corresponds to a statistically significant *c-SKL*. As only datasets within the same platform can be compared, i.e., datasets measuring the same sets of variables, a different network is constructed for each platform. The network for GPL570 and all statistically significant edges is shown in Fig. 3a; the networks for the other platforms are found in SM5. Figure 3b depicts the same network, when only the top 300 most statistically significant edges are included. In the figure, several communities emerge (a community is defined as a set of nodes densely interconnected). Manually inspecting and annotating the nodes provides evidence for the efficacy of the method: in Fig. 3b, we observe that datasets within a community typically pertain to the same disease and/or tissue, which could explain the similarities found. Missing edges are potentially interesting and informative too: the GSE20036 dataset was one of the few that had no edge to any other dataset for the GPL570 platform and is thus not shown in the network. On closer inspection, the dataset refers to gene expression analysis of deer antler measured using human micro-arrays which explains a high *c-SKL* with all the other human datasets. There are two communities of breast cancer shown that are not connected; on closer inspection the largest one contains case-control studies on human subjects, while the other contains treatment-control studies on cell lines, justifying why the *c-SKL* is high among studies belonging in these two different groups. It is well known that significant differences between cell lines and human tumors exist.^3^

It is also interesting to study the evolution of these networks, e.g., using the on-line app provided, as one varies the number of edges to include. In general, as we increase the number of edges in the network, similar communities tend to connect and merge together. For example, if we go beyond the 300 edges shown in Fig. 3b, we will observe the brain diseases’ community merging with the glioma community and the several lymphoma related datasets to connect with each other. Some more snapshots of the networks for different number of edges and platforms are shown in SM5.

### From dataset similarity to disease similarity

**Fig. 4:**
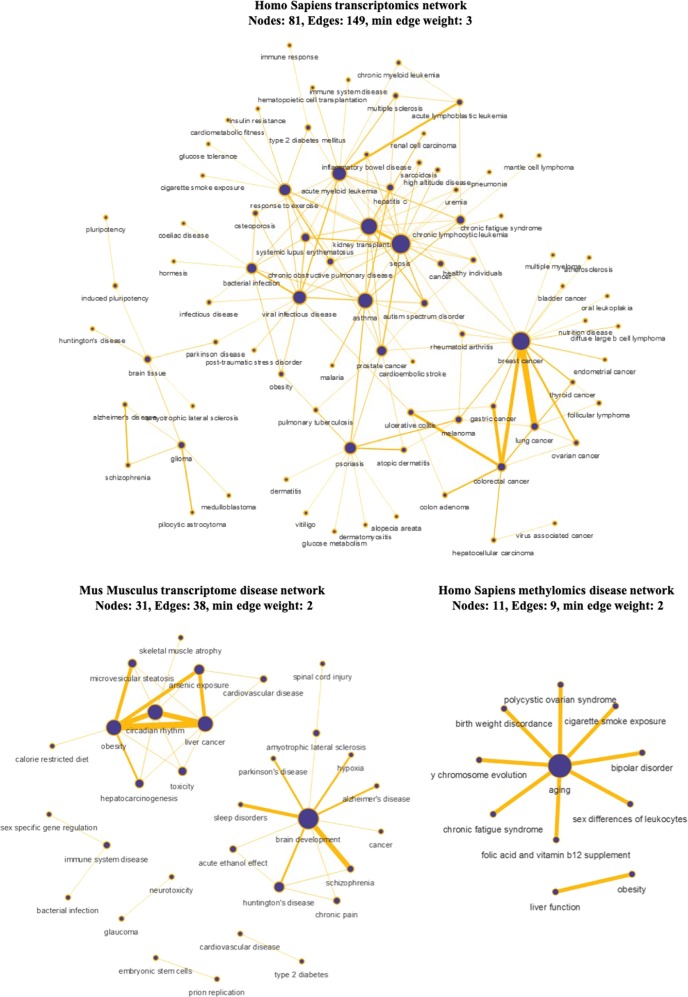
Disease networks of Homo Sapiens and Mus Musculus. Nodes represent diseases or phenotypes (i.e., response to exercise). Edge weights (denoted with the edge thickness) represent the number of times (number of dataset pairs) the corresponding disease-disease relation has been found. Node size increases with the number of neighbors

Even though the proposed *c-SKL* metric cannot distinguish among the sources of observed similarities, yet, it can point-out unexpected similarities. For example, the similarity networks (SM 5) demonstrate that tissue is not the only reason of similarity, as we notice significant similarities in datasets measuring different tissues (i.e., anterior orbit or lacrimal gland and blood). At the same time, not all datasets providing data from the same tissue interconnect, e.g., datasets of blood tissue measurements do not fully interconnect. To systematically extricate non-trivial and potentially interesting similarities, we focused on the similarities that connect macroscopically different diseases. We construct a new type of network where each node corresponds to a different disease, or phenotype in a more general sense, a disease-to-disease network: two nodes *A* and *B* in this network are connected by an edge (*A,B)*, whenever a dataset of disease *A* is found similar to a dataset of disease *B*. We construct one disease-to-disease network for each different species (Homo Sapiens and Mus Musculus) and each type of -omics (transcriptomics and methylomics) by assembling all edges from all related platforms. Figure 4 shows the disease-to-disease network for Homo Sapiens transcriptomics (both micro-array and RNA-seq based), Homo Sapiens methylomics as formed by similarities based on DNA methylation levels of >450,000 CpG methylation sites and Mus Musculus transcriptomics. The networks depict disease similarities at the molecular level. The methylomics disease network is much smaller, as there are only 117 datasets from the GPL13534 platform. Edges *(A, B)* in Fig. 4 are annotated with a weight, corresponding to edge thickness, equal to the number of times a pair of studies one from disease *A* and one from *B* are found similar. Thus, edges of high weight indicate a molecular similarity between different diseases found multiple times across studies and platforms and that could be attributed to an underlying common biological mechanism. Figure 4 (top) for human transcriptomics shows only the edges of weight 3 and above, while Fig. 4 (bottom right) for methylomics shows edges of weight 2 and above, as methylation datasets are much fewer and edge weights are lower. Figure 4 (bottom left) shows Mus Musculus transcriptomics from the GPL1261 platform.

Focusing on Fig. 4 (top), the strongest edge with weight of 34 is found between breast cancer and lung cancer. The finding provides additional evidence to the literature: it has been recently reported that lung tumors express estrogen receptor alpha, which is a common trait in >70% of breast tumors, and whose activation expresses pathways related to cell proliferation and metastasis,^4^ common elements shared with pathways identified in breast cancer.^5^ Also, a connection between breast cancer and thyroid cancer appears (weight = 7), a relationship that has attracted substantial attention in the medical research community.^6^ Alzheimer’s disease and schizophrenia, a similarity found in eight different data-pairs, is known to share psychiatric symptoms that suggest some common cerebral pathophysiology.^7^ In,^8^ a network that spatially recapitulates the pattern of brain abnormalities observed in both schizophrenia and Alzheimer’s disease was revealed by a data driven analysis. Psoriasis and asthma (weight = 6), both chronic immune-mediated inflammatory diseases, is another example of a rather unexpected similarity, which has attracted much attention recently.^9^ Melanoma and breast cancer, an already known similarity^10–12^ also appears frequently (weight = 5). Other interesting, frequent similarities that can be further supported by evidence found in the literature, include the links between acute myeloid leukemia and viral infectious diseases (weight = 7),^13^ asthma and malaria (weight = 5),^14^ asthma and hepatitis C (weight = 6),^15^ systemic lupus erythematosus and viral infectious disease (weight = 6),^16^ or Parkinson’s disease and Alzheimer’s disease (weight = 2, out of the only two available Parkinson’s disease datasets).^17^ It is also interesting to note that in the Mus Musculus transcriptome network the most frequent similarity was found between obesity and liver cancer (weight = 6). See ref. ^18^ for a review on the existing evidence on the links between obesity and liver cancer incidence and survival and^19^ for a pooled analysis from all U.S. based studies in the NCI Cohort Consortium. For a ranked list of all disease associations that have been found at least four times in any of the Homo Sapiens platforms see Table 2 in SM6.

### Uncovering molecular underpinnings of statistical similarities

**Fig. 5:**
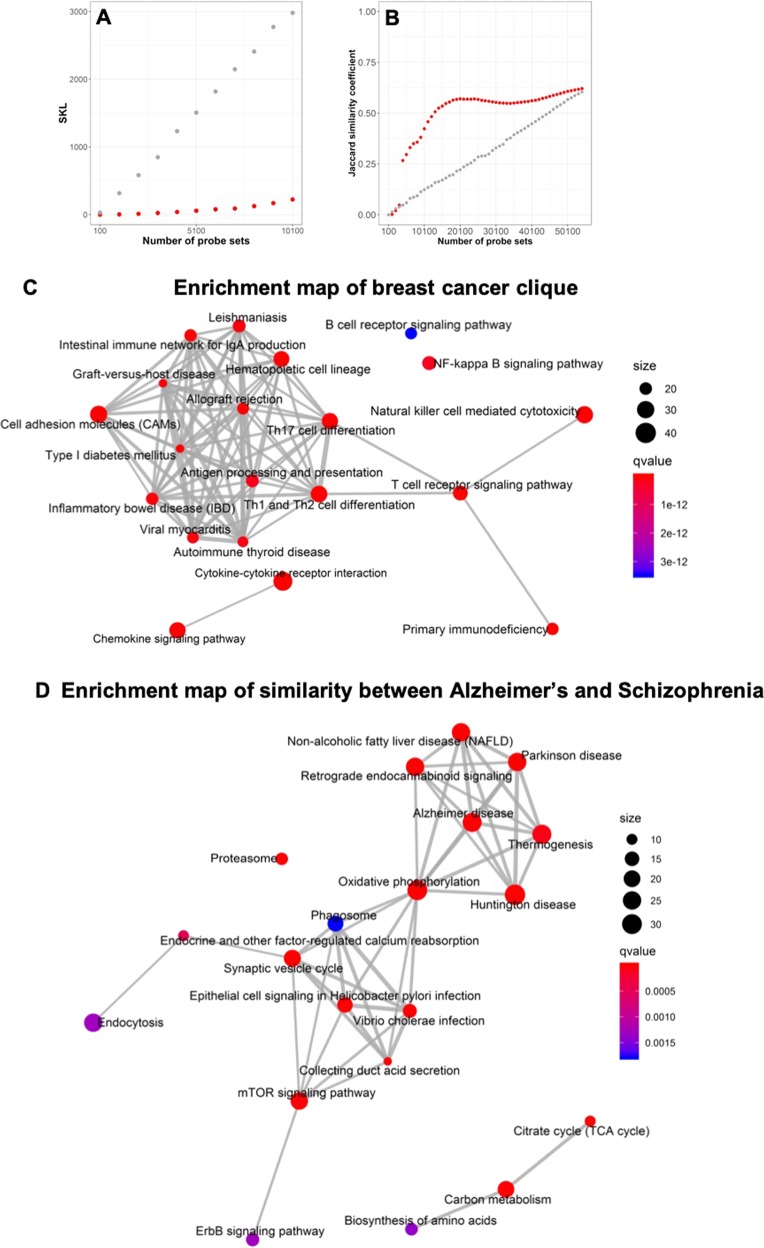
Statistically and biologically explaining a high-dimensional curated Symmetric Kullback–Leibler Divergence. **a** *c-SKL* versus the number of top *k* probe sets that best explain the similarity of two datasets (GSE37171 and GSE46474 measured by GPL570) is shown in red. Gray color corresponds to the *c-SKL* computed using the same number of randomly selected probe sets. **b** Jaccard similarity coefficient between two cliques of AML measured by different platforms, GPL570 (microarray) and GPL11154 (RNA-seq) is shown in red. Gray color corresponds to the Jaccard index of the same similarity computed based on a random selection of genes. **c**, **d** Enrichment maps of the 20 most statistically significantly enriched pathways by the genes that explain a breast cancer clique of ten datasets measured by GPL570 and the similarity of eight different pairs of datasets connecting Alzheimer’s disease with schizophrenia

Once an interesting similarity in the dataset-to-dataset network has been identified, it becomes important to explain it in a way that conveys biological insight and understanding. The above methods and ideas are exemplified in Fig. 5. Specifically, we consider the similarity between datasets GSE37171 and GSE46474 measured by GPL570. This is the most statistically significant similarity found in any pair of datasets examined. In Fig. 5a we show that the *c-SKL* achieved using the *B(k)* features (shown in red), obtained by the proposed optimization method that explains *c-SKL* (see Methods), dominates (obtains a lower *c-SKL*) the *c-SKL* achieved by randomly selecting *k* features (gray color). The graph starts at value *k* *=* 100 with a step size of 1000. More examples are shown in SM4.1, demonstrating that the curves are qualitatively similar over the whole range of similarities and platforms. The results clearly show that *B(k)* selects better explaining features than random guessing. Next, we consider the hypothesis that, for the same disease, the biological mechanisms that best explain similarities between datasets at the transcriptome level, should be common, independently of the measuring technology. As an example, we first identified an acute myeloid leukemia (AML) clique of five datasets measured by GPL570 (microarray gene-expression) and an AML clique of five datasets measured by GPL11154 (RNA-seq) and compared the gene sets that explain their similarity (see Fig. 5b). The Jaccard index when including all ~50000 probe sets measured by both platforms is 0.62. This number is closely approximated when the number of best explaining probe sets is 15,000–20,000. The gray dots correspond to the Jaccard index of random selection of probe sets between the two platforms for comparison. A qualitatively similar plot for another example on psoriasis can be found in SM8. These two examples illustrate that the lists of genes explaining the similarities among datasets of the same disease are similar across measuring platforms.

Finally, we show two examples of the ability of the method to convey biological intuition in the form of a set explanation. Specifically, we simultaneously explain using 1000 probe-sets, (a) all 45 pair-wise similarities found within a clique of ten breast-cancer datasets and (b) eight similarities of Alzheimer’s disease to schizophrenia. In both cases, the datasets are gene-expression micro-arrays measured by GPL570. The probe-sets are then mapped to their corresponding gene symbols and an enrichment analysis of KEGG pathways is performed. Figure 5c, d visualizes the enriched pathways as enrichment maps. Regarding (a), the map (Fig. 5c) for explaining the breast cancer clique shows several immune related pathways that verify the already known immune system tumor infiltration related to breast cancer evolution (see SM7 for a detailed discussion). A similar example and discussion on a lung cancer clique is shown in SM7. Regarding (b), the pathways (Fig. 5d) that explain the similarity of Alzheimer’s disease to schizophrenia include six out of the ten KEGG pathways that are related to the nervous system.

## Discussion

The traditional disease classification system (e.g., ICD^20^) groups diseases with similar clinical symptoms and phenotypic traits. One of the first attempts to group diseaes with common genetic origin was the work by Goh et al.,^21^ who created the Diseasome, a visual reference of the genetic links between disorders and disease genes. The human disease network they created, displayed many connections between both individual disorders and disorder classes and proved that studying diseases and phenotypes from a systems approach offers the possibility to discover general patterns and principles of human disease. Since then, several network based approaches to understand the molecular basis of human disease, appeared. Hidalgo et al.,^22^ for example, exploited disease phenotypes of more than 30 million elderly Americans aged 65 or older, to create a Phenotypic Disease Network (PDN) where nodes represented disease phenotypes, as defined by the ICD9 and edges a significant comorbidity according to the measures they introduced. Later, Žitnik et al.^23^ found relationships between diseases, some of them undiscovered at that time, by fusing molecular interaction and ontology data from several biological databases, such as Gene Ontology, Disease Ontology, DrugBank and others. Sun et al.^24^ estimated the similarity score of diseases, by analyzing four publicly available disease-gene association datasets (OMIM, CTD, FunDO, and HuGENet) and demonstrated their similarity measure by identifying diseases associated with diabetes mellitus which were further confirmed bibliographically. Yang et al.^25^ proposed a more data driven approach, by applying differential co-expression analysis to explore the architecture of disease relationships in terms of dysfunctional regulation mechanism. Their results proved that their approach can be a complement to the disease networks generated from symptoms, disease concepts and biomedical data. Later, Menche et al.^26^ identified common mechanistic pathways between diseases by exploiting the overlap of disease modules (connected subgraphs formed by the interaction of disease proteins). Recently, Halu et al.^27^ developed and analyzed the human disease multiplex network by considering genotypic and phenotypic information simultaneously and showed, among other results, that Mendelian disorders predispose individuals to more common, complex diseases. Other approaches that compare biological datasets rely on creating a molecular signature list for each dataset containing the differentially expressed quantities with respect to a target variable (supervised approach). A typical such target variable in case-control studies is the disease status, and in treatment-control studies it is the presence or absence of treatment. Dataset similarities are then computed as similarities between the signature lists.^28–30^

The above studies do not take advantage of the enormous and rapidly growing public repositories of omics data or may require phenotypic annotation and labeling of samples and datasets (supervised approaches). The latter is technically difficult to perform automatically and scale up to thousands of datasets. Instead, in this work, we propose a method that is unsupervised and depends only on the omics data distributions. Hence, it can easily be applied to the entire collection of available omics datasets.

The proposed method could also be employed for what we call *data-based* information retrieval, i.e., retrieving all datasets that are similar to a particular query dataset (e.g., return all public mice datasets with molecular patterns similar to a users’ mice profiles under a specific new treatment). Data with similar statistical properties as the query data could be potentially pooled together for meta-analysis or identify similar control samples from totally unrelated studies. It is worthwhile to note that quite recently, Google launched a new service, called Dataset Search, also aiming at dataset retrieval. However, Dataset Search performs retrieval based on the metadata tags, while the proposed method identifies statistically similar datasets independently of any text or tag annotations.

As most human public -omics datasets are case-control studies, in this work we focused on disease-to-disease similarities; however, in the context of drug design, one could examine treatment-control studies to create treatment-to-treatment networks depicting similarities between expression patterns induced by different compounds. Compounds, tested for different diseases that yet induce similar expression patters for unexpected reasons, are prime candidates for drug repurposing.

If one annotates datasets with the tissue measured (or some other datasets characteristic), then it is possible to estimate the principal component corresponding to the tissue and remove it. Recomputing similarities after this removal will highlight similarities due to all other reasons except tissue, further revealing common biological mechanisms among different diseases.

Finally, while the proposed method requires two datasets to be measuring the same set of variables (molecular quantities), it is possible to develop methods of comparing distributions over different sets of variables (human genes vs. mice genes), an interesting line of future work that we are actively exploring.

## Methods

### Comparing statistical distributions of high-dimensional, low-sample datasets

Two datasets can be viewed as two dimensional matrices *DS*_*P*_ and *DS*_*Q*_ with rows ranging over the samples and columns over the variables (features, molecular quantities measured, attributes; e.g., in the context of gene-expression micro-arrays variables are probe sets). For -omics data, such as micro-array or RNA-seq data, the number of variables ranges into the tens to hundreds of thousands, while often the sample size is less than a few dozens. To compare such high dimensional, low-sample size empirical distributions, we propose the following methodology: First, each dataset is standardized by subtracting the mean and dividing by the standard deviation of a variable for each measured value. Standardization removes the effect of the mean and the scale of the measurements, which could depend on the sensitivity of the measuring instrument and the concentration of the reagents and may be sensitive to other nuisance factors. This transformation retains the linear correlations among variables. Second, each dataset *DS*_*P*_’s distribution is assumed to be multivariate normal and thus fully determined by its mean (which is transformed to zero) and its symmetric, positive semidefinite covariance matrix *Σ*_*P*_ (coinciding with the correlation matrix for standardized data). Given that the number of variables is larger than the number of samples, the sample covariance matrix is rank deficient. A third assumption (which enables the estimation of the rank-deficient covariance matrix) is that Σ_*P*_ can be expressed as Σ_*P* _= *P*Λ^*P*^*P*^*T* ^+ *σPI*, where *P*_*n×k*_ is an orthonormal matrix, *Λ*^*P*^_*k×k*_ a diagonal matrix with the *i*_th_ value in the diagonal denoted as $\lambda _i^P \ge 0$, and *σ*_*P*_ a non-negative real. In other words, it is assumed that the data lie in a *k << n* dimensional subspace, where *n* is the number of variables, with an isotropic noise component (see SM2.1). The decomposition of *Σ*_*P*_ is computed using the standard Principal Component Analysis. Alternatively, one could employ Probabilistic PCA to directly compute the maximum likelihood decomposition of *Σ*_*P*_^31^ at the expense of higher computational cost. *P* is the matrix of the first *k* *=* *c*_*P*_ principal axes of the sample covariance matrix, $\lambda _i^P$ the corresponding eigenvalues ($\sqrt {\lambda _i^P}$ are the singular values of *DS*_*P*_). Similarly, for dataset *DS*_*Q*_ we compute the decomposition Σ_*Q*_ = *Q*Λ^*Q*^*Q*^*T*^ + *σ*_*Q*_*I*, where *Q* is the matrix of the first *c*_*Q*_ principal axes and Λ^*Q*^ contains the *c*_*Q*_ eigenvalues of the covariance matrix in its diagonal. Based on the above ideas, we develop a measure of (dis)similarity between the distributions of the two datasets *DS*_*P*_ and *DS*_*Q*_, called the curated symmetric Kullback–Leibler (*c-SKL*) divergence, and denoted as *c*-*SKL* (Σ_*P*_, Σ_*Q*_). This measure depends only on the covariance matrices given the above assumptions. The smaller the *c-SKL* between a pair of datasets, the more similar the two distributions and their covariance matrices are (Fig. 2b)*. c-SKL* is symmetric and non-negative, but the triangle inequality does not necessarily hold, so it is not a proper metric.^32^ Other similarity metrics for high-dimensional, low-sample settings could be employed, such as the Maximum Mean Discrepancy or MMD.^33^ However, the proposed *c-SKL* depends on a PCA decomposition that can be explainable, as we later demonstrate; in addition, the PCA decomposition can be pre-computed and stored for each dataset to be employed for all subsequent pairwise *c-SKL* computations. This type of caching makes *c-SKL* computations efficient for applications when the collection of datasets is constantly updated. Based on the assumptions above, for two datasets *DS*_*P*_ and *DS*_*Q*_, where σ ≈ σ_P_ ≈ σ_Q_ the *c-SKL* can be approximated as:

1 $$\overline {c-SKL} _{\left\langle a \right\rangle }\left( {{\mathbf{\Sigma }}_P,{\mathbf{\Sigma }}_Q} \right) \approx \frac{1}{{2(1 - a)}}\left[ {2{\mathrm{an}} - {\sum\limits_{i = 1}^{c_P}} {{\sum\limits_{j = 1}^{c_Q}} {\lambda _i^P} } \left( {P_i^T \cdot Q_j} \right)^2 -{\sum\limits_{i = 1}^{c_P}} {{\sum\limits_{j = 1}^{c_Q}} {\lambda _i^Q} } \left( {P_i^T \cdot Q_j} \right)^2} \right]$$

Equation (1) is an approximate version of Eq. 26 shown in SM 2.2. Both equations are proved in SM 2.2. Equation (1) has an intuitive geometric interpretation: the first double sum computes the squared length of the projections of $\sqrt {\lambda _i^P} P_i$ onto the subspace spanned by the principal axes of *Q*. The reverse is true for the second double sum. Obviously, the *c-SKL* divergence is minimized when the two subspaces coincide. The empirical success of the formula depends on the choices of the number of principal axes to retain *c*_*P*_ and the value of *σ*_*P*_ in each decomposition ${\mathrm{\Sigma }}_P = P{\mathrm{\Lambda }}^PP^T + \sigma _PI$. Selecting the first *c*_*P*_ principal axes to retain explains $\mathop {\sum}\nolimits_{i = 1}^{c_P} {\lambda _i^P}$ of the total dataset variance *n* (since there are *n* variables, each with variance and standard deviation of 1). The value of *c*_*P*_ is determined in such a way that the first term explains just about *α*_*P*_ percent of the variance, i.e., $c_p = {\mathrm{argmin}}_{\left\{ {c \in N} \right\}}\left( {\mathop {\sum}\nolimits_{i = 1}^c {\lambda _i^P} \ge \alpha _Pn} \right)$. The parameter *α*_*P*_ dictates the level of compression of the data. As *α*_*P*_ increases, more principal axes are employed, enlarging the axes subspace and allowing finer differences (dissimilarities) to other datasets to be discovered; at the same time, as *α*_*P*_ increases the additional axes entering the equation are less reliably estimated. The parameter *α*_*P*_ is the variance-per-variable unexplained by the principal components and distributed to the isotropic noise components. We set it to 1 − *α*_*P*_ so that the sum of variances in the decomposition equals the variance of the original dataset. Employing the same value for *α*_*P*_ in all *c-SKL* computations among different pairs of datasets (leading to drop the index *P* from the notation), makes two *c-SKL* values comparable as they are all computed using the same compression level. In fact, after experimentation we found that results improve when eigenvalues are scaled so that they sum exactly to *α*, i.e, $\mathop {\sum}\nolimits_{i = 1}^{c_P} {\hat \lambda _i^P} = \alpha$, where $\hat \lambda$ are the normalized values (the method is graphically depicted and explained in SM section 2.3–2.4 and Supplementary Fig. 3). In order to statistically validate the method, we performed the following computational experiment: We randomly partitioned each dataset, with respect to samples, into two “sibling” datasets *DS*_*1*_ and *DS*_*2*_. We computed *c-SKL* among each pair of all available datasets, agnostic to the above partitioning. By construction, the sibling datasets come from the same distribution and should be discovered to be closer in *c-SKL* than with any other dataset. The validation experiment and its results are presented in detail in SM3, where we show that the sibling of a dataset is found closer in terms of *c-SKL* than any other dataset 95% of times. From the experiment we also determined a reasonable value for *α* to be 50%, but results are robust with respect to the specific numerical value used. We then estimate the statistical significance of similarities and keep only the ones with a *q*-value (adjusted *p*-value for multiple testing based on the Benjamini and Hochberg procedure^34^) less than 0.05 using a semi-parametric bootstrap technique. The semi-parametric bootstrap testing procedure is as follows: Let *J* be the distribution of the pooled samples across all datasets; let *P* and *Q* be the distributions of two datasets *DS*_*P*_ and *DS*_*Q*_. The alternative hypothesis tested is that the observed *c-SKLs* between *P* and *Q* is smaller (and thus their similarity is larger) than the *c-SKLs* of *P* and *J*, as well as *Q* and *J*. In other words, a statistically significant similarity indicates that two datasets are more similar to each other, than with the rest of the samples (see also SM 2.4). We note that, there exist procedures in the literature testing the exact equality of high-dimensional distributions,^33,35^ i.e., H_0_: *P* *=* *Q* or equivalently, H_0_: *s* *=* *0*, where *s* is their *c-SKL*. However, in the context of this work, the null hypothesis of exact equality of distributions is meaningless and should typically be rejected; studies may be related, but do not necessarily employ exactly the same population under the same experimental and sampling conditions. Instead, we propose to test for a relatively high similarity with respect to the rest of the samples in the pooled collection of all datasets.

### Explaining a curated Symmetric Kullback–Leibler Divergence

It is not straightforward to explain why two distributions over tens of thousands of dimensions are similar. As an explanation, we propose to report the top *k* molecular quantities, to which the similarity can be mostly attributed. As proved in SM2.2 (Lemma 2), a low Kullback–Leibler divergence corresponds to a similarity between the corresponding covariance matrices *Σ*_*P*_ and *Σ*_*Q*_. The most contributing features correspond to the rows and columns of the covariance matrices, where the matrices agree the most (see Fig. 2d and SM section 2.5 for a discussion). The set of best “explaining” features can then be examined for enrichment of pathways, ontologies, or other predefined interesting groups of molecular entities. To find the top *k* best explaining index set *B* (for Best) given two datasets with principal axes in *P* and *Q*, and eigenvalues in vectors *λ*^*P*^ and *λ*^*Q*^ one needs to solve the following optimization problem:

2 $$B\left( k \right) = {\mathrm{argmin}}_{S}\frac{1}{{2\left( {1 - a} \right)}}\left[ {2{\mathrm{an}} -{\sum\limits_{i = 1}^{c_P}} {{\sum\limits_{j = 1}^{c_Q}} {\left( {\lambda _{i}^{P} + \lambda _{j}^{Q}} \right)} } \left( {P_{i}^{T}{\mathrm{diag}}\left( S \right)Q_{j}} \right)^{2}} \right],$$

such that *S* is a vector of exactly *k* ones and everything else zero, and diag*(S)* produces a diagonal matrix with *S* on the diagonal. The *S* vector serves as the selector of features in the computation of Eq. (1). The parameter *k* is arbitrarily selected by the user. This is a quadratic, constrained optimization problem that can efficiently be solved approximately by considering its bilinear form:

3 $${\mathrm{argmax}}_{T,S}\left[ {{\sum \limits_{i = 1}^{c_P}}{\sum \limits_{j = 1}^{c_Q}} C_{ij}\left( {P_{i}^{T}{\mathrm{diag}}\left( T \right)Q_j} \right)\left( {P_{i}^{T}{\mathrm{diag}}\left( S \right)Q_{j}} \right)} \right].$$

where $C_{ij} = (\lambda _i^P + \lambda _j^Q)$. A simple algorithm for solving (3) starts with an initial value for *S* and alternating between solving for vectors *T* and *S* until convergence when *T* *=* *S* = *B*. Solving for *T* considering *S* fixed and vice-versa is a linear, constrained problem that can be solved trivially (see SM section 2.5 for more details). In addition to identifying the indexes of the *k* best-explaining features *B*(*k*), one can instead minimize the equation above to find the top *k* features that make the distributions most different, denoted by *W*(*k*) (standing for *W*orst).

As presented, *B*(*k*) explains a single dataset-to-dataset similarity; however, one could extend this method to find the features that simultaneously best explain a set of pair-wise dataset similarities, trying to pick the common biological mechanisms that are jointly responsible for all similarities observed in the set. To find the best set explanation among a set of pair-wise similarities, we solve a similar optimization problem maximizing the sum of all pair-wise *c-SKLs* within the set (see SM2.5 for an efficient implementation of this algorithm). There are at least two scenarios where a set explanation could be useful. For example, one may wish to explain all pair-wise similarities among datasets pertaining to the same disease. These datasets may differ slightly in terms of patient population, experimental conditions, and sampling methodology; identifying the genes that explain the patterns shared by all of them, accentuates the common cause for the similarities in the group, presumably those genes involved in the common disease mechanism. A second use of a set explanation is when trying to explain an edge in the disease-to-disease network. Each such edge corresponds to a set of similarities whose set explanation sheds light to the common mechanism involved in making the two different diseases similar.

### Reporting summary

Further information on research design is available in the Nature Research Reporting Summary linked to this article.

## Supplementary information

A data driven approach revealing disease similarity at the molecular level_SM

Reporting summary

## Data Availability

All datasets used in this work are available in: http://dataome.mensxmachina.org/.

## Code availability

Any code used in this work can be accessed, upon request to the corresponding author.
