# Supplementary material for: A data driven approach reveals disease similarity on a molecular level
Source: NPJ Syst Biol Appl. 2019 Oct 25;5:39. doi: 10.1038/s41540-019-0117-0 (PMC6814739; doi:10.1038/s41540-019-0117-0)
Supplement: Supplementary file 1 — A data driven approach revealing disease similarity at the molecular level_SM [file 41540_2019_117_MOESM1_ESM.pdf]

Supplementary Material

A data driven approach revealing disease similarity on a molecular level

Kleanthi Lakiotaki*, George Georgakopoulos,
Elias Castanas, Oluf Dimitri Røe, Giorgos Borboudakis, Ioannis Tsamardinos*.

Contents

1 Materials....4
2 Supplementary Methods....7
3 Validating the *c-SKL* method: The "siblings" experiment....21
4 Validating the method that explains the SKL method based on enriched pathways....24
5 Visualizing the Landscape of -omics data....27
6 From dataset similarity network to disease similarity network....35
7 Molecular underpinnings of breast and lung cancer....42
8 Comparing findings from different measurement technologies....51
SUPPLEMENTARY REFERENCES....53

List of Figures

TaSupplementary Figure 1: Disease/ phenotype distribution of all samples across different technologies....5
Supplementary Figure 2: Sample size distribution of datasets across the different measurement technologies....6
Supplementary Figure 3: A schematic representation of the KL-divergence between two statistical distributions....18
Supplementary Figure 4: Pseudocode of the "siblings" experiment....22
Supplementary Figure 5: Frequencies of the rank of sibling datasets for each measurement technology....23
Supplementary Figure 6: Validating the explain c-SKL heuristic method....26
Supplementary Figure 7: Bar plot of the number of statistically significant connections of each dataset (a.k.a. node degree in network science) for each measurement technology....28
Supplementary Figure 8: Frequency of identical phenotype to phenotype similarities....36
Supplementary Figure 9: Disease to disease network....38
Supplementary Figure 10: Dotplot of the results of Gene Ontology overrepresentation analysis of breast cancer gene sets....46
Supplementary Figure 11: KEGG Allograft Rejection pathway in the lung cancer clique of 9 datasets....50
Supplementary Figure 12 Left. Jaccard similarity coefficient between two cliques of AML measured by GPL570 and GPL11154. Right. Jaccard similarity coefficient between two cliques of psoriasis measured by GPL570 and GPL11154....52

List of Supplementary Tables

Supplementary Table 1: Symbolism used in mathematical grounds of the proposed dataset similarity method....8
Supplementary Table 2: Disease to disease similarities found in Homo Sapiens gene expression datasets....40
Supplementary Table 3: Top 20 most significantly enriched pathways that explain the breast cancer clique....46
Supplementary Table 4: Top 20 most significantly enriched pathways that explain the lung cancer clique....49

54

1 Materials

1.1 Public biological data repositories

A subset of BioDataome, a collection of uniformly preprocessed and automatically annotated datasets for data-driven biology [1], including ~5600 datasets of ~260000 samples spanning ~500 diseases was used in this work. This subset includes 978 datasets from six different -omics measurement technologies (five transcriptomics and one epigenomics). The datasets were selected with the following criteria:

1) They include at least 40 samples. The exact threshold of 40 was chosen arbitrarily as a minimum threshold to obtain statistically reliable results in distributions of about 50000 or even 500000 dimensions (molecular quantities measured).
2) They share no common samples, as this may lead to similarities identified due to the common samples and not their molecular underpinnings that may reveal interesting biology.
3) They require at least three principal components that explain at least 50% of the variance, as a result of the principal component analysis that we perform as a first step for the proposed method (see section 2).

In BioDataome, all datasets are automatically annotated with a disease term from the Disease-Ontology [2]. Here, we further manually checked these automatically assigned disease terms for possible mislabels and also assigned either a disease, or a phenotype term (i.e. GSE11761 studies "response to exercise") to any unlabeled datasets. The distribution of the disease/ phenotype of the datasets is shown in Supplementary Figure 1. Among all diseases "breast cancer" and 'lung cancer' enjoy the maximum share. In Supplementary Figure 2 we show the sample size distribution of datasets from all measurement technologies. Not surprisingly, most datasets have relatively low (<150) sample size. This corroborates our intuition that any similarity-identifying method should be reliable to low-sample, high-dimensional settings in order to be employed for the creation of the landscape of the biological dataome.

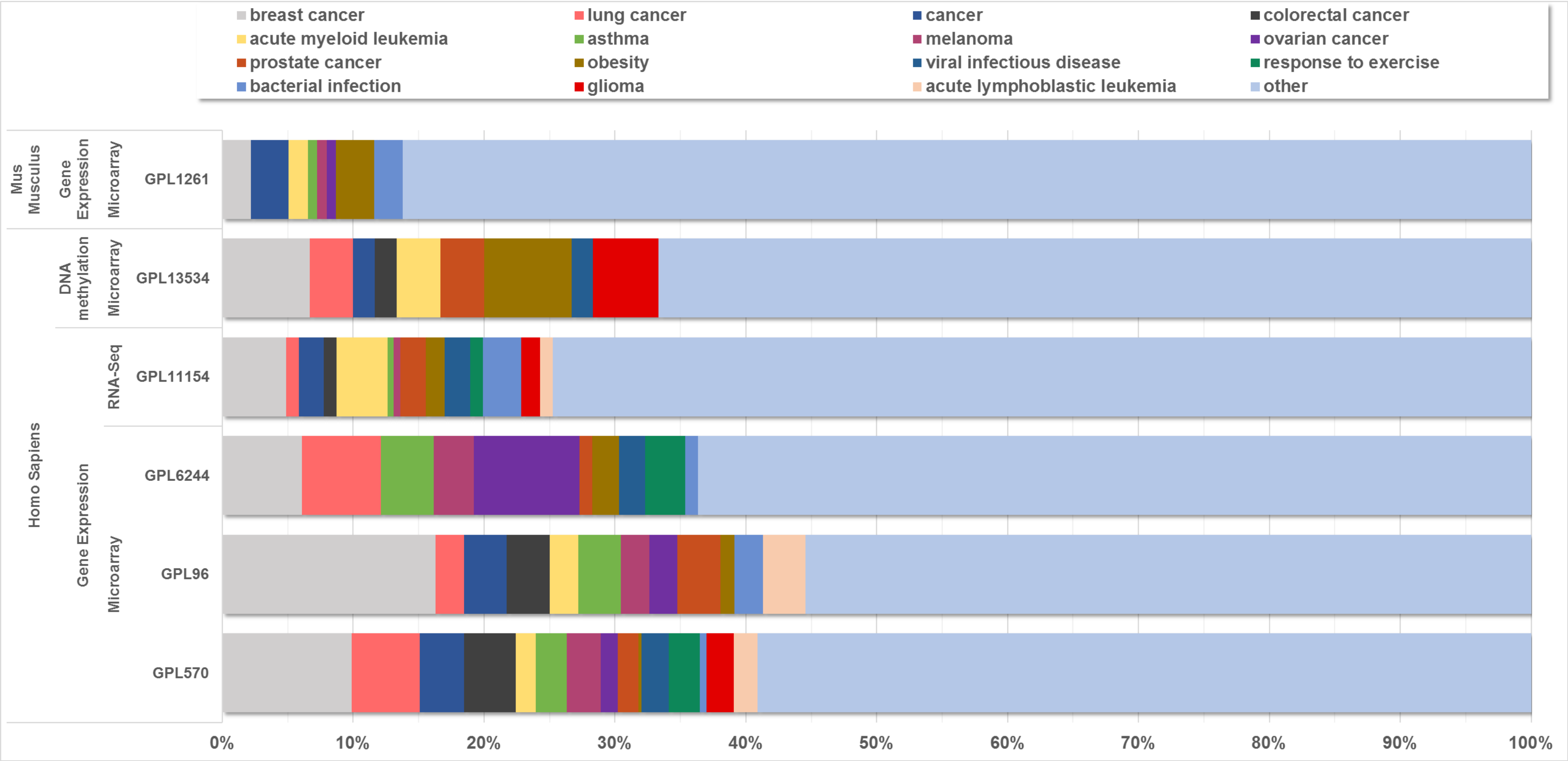

Supplementary Figure 1: Disease/ phenotype distribution of all samples across different technologies. First 15 most frequent diseases are shown. All other diseases are grouped together (light blue bars).

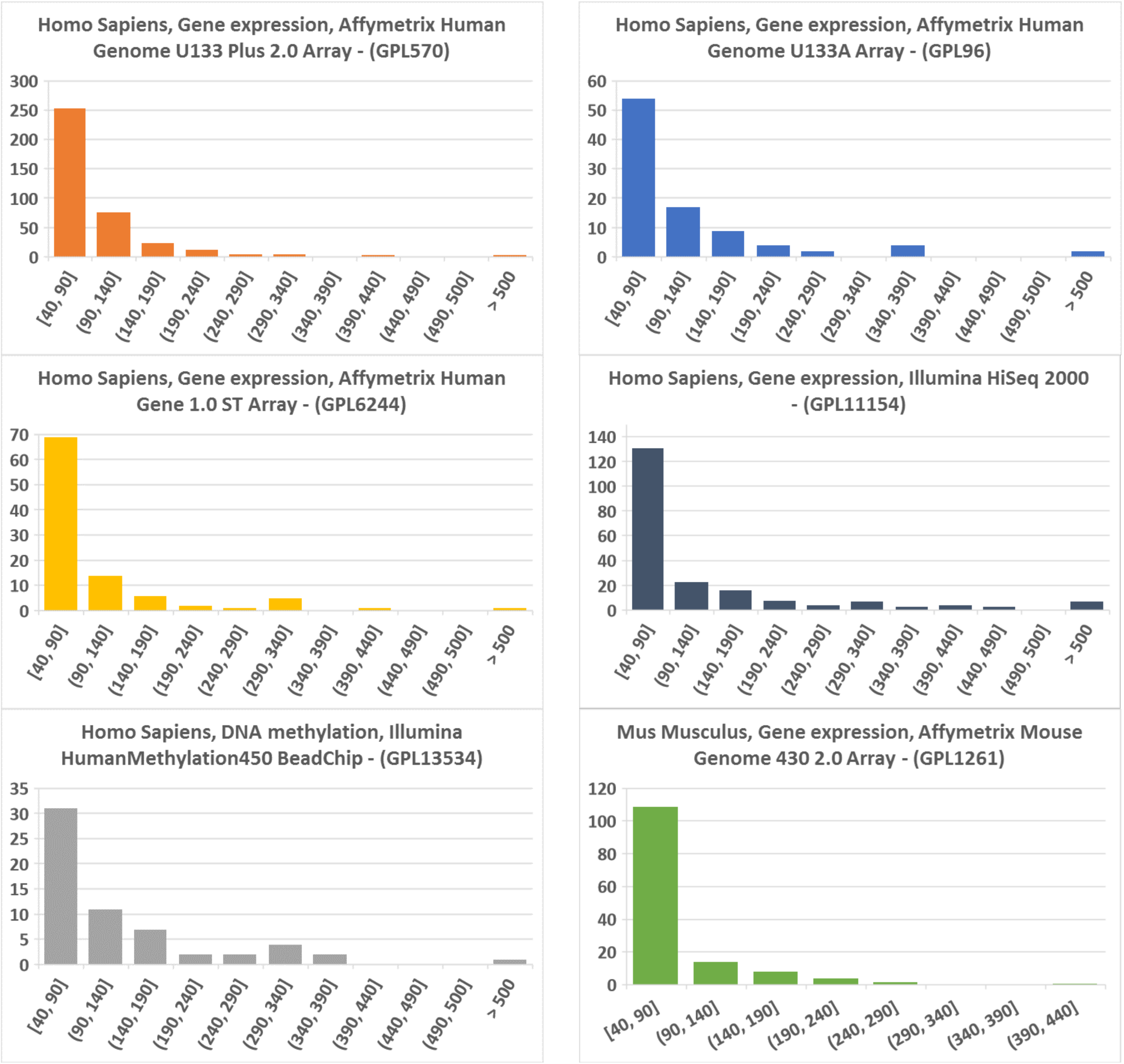

84 *Supplementary Figure 2: Sample size distribution of datasets across the different measurement technologies. X axis represents*
85 *sample size groups and Y axis their respective counts.*

2 Supplementary Methods

2.1 Foundations of informational similarity

Here we provide the mathematical grounds on which the data manipulations and experiments of the main text are based. The core object of our investigation is the similarity between a pair of datasets, each consisting of a number of observations drawn from two statistical populations, named 'P' and 'Q'. The data obtained, are tabulated as tables $\boldsymbol{DS}_P[1 \dots s_P, 1 \dots n]$ and $\boldsymbol{DS}_Q[1 \dots s_Q, 1 \dots n]$, where s_P and s_Q are respectively the number of observations (also referred as samples) taken from the populations 'P' and 'Q', and n is the number of variables (molecular quantities) measured.

One measure of informational similarity is the *Kullback-Leilber divergence*. For two probability densities p and q over the same event space Ω it is defined [3] as:

$$\boldsymbol{KL}(\boldsymbol{p}||\boldsymbol{q}) \stackrel{def}{=} \int_{e \in \Omega} \boldsymbol{ln}\left(\frac{\boldsymbol{p}(\boldsymbol{e})}{\boldsymbol{q}(\boldsymbol{e})}\right) \boldsymbol{p}(\boldsymbol{e}) \quad (1)$$

The lower the KL, the more similar are the two distributions. A main problem in order to make this similarity measure applicable within our framework is that the sample size is usually very small with respect to the number of variables measured. The number of variables range in the tens or hundreds of thousands, while the sample size is most often less than 150. This fact renders rather meaningless any attempt to directly estimate the actual probability distributions $p(\cdot)$ and $q(\cdot)$.

To address this problem, we propose the following:

a) We assume the probability distributions of 'P' and 'Q' are standardized, multivariate normal distributions, i.e, they follow distributions $N(\boldsymbol{0}, \boldsymbol{\Sigma}_P)$ and $N(\boldsymbol{0}, \boldsymbol{\Sigma}_Q)$, respectively, where $\boldsymbol{\Sigma}_P$ and $\boldsymbol{\Sigma}_Q$ are the covariance matrices.

b) The covariance matrix for distribution 'P' can be decomposed as $\Sigma_P = P\Lambda^P P^T + \sigma_P I$, where $P_{n\times k}$ is an orthonormal matrix, $\Lambda^P{}_{k\times k}$ a diagonal matrix with the i_{th} value in the diagonal denoted as $\lambda_i^P \geq 0$, and σ_P a positive real. In other words, it is assumed that the data lie in a $k << n$ dimensional subspace, where n is the number of variables, with an isotropic noise component. This procedure adds $+\sigma_p^2\boldsymbol{I}$ to the diagonal arguments and makes Σ_P full rank, positive definite, and thus invertible. Similarly, $\Sigma_Q = Q\Lambda^Q Q^T + \sigma_Q I$, where Q is an orthonormal matrix and Λ^Q a diagonal matrix.

Within the rest of this section we will use the symbolism provided in Supplementary Supplementary Table 1 and present the implementation of the above ideas.

Definition	**‘P’**	**‘Q’**
Datasets	$\boldsymbol{DS}_P$	$\boldsymbol{DS}_Q$
Dimension (number of variables)	n	n
Number of samples	s_P	s_Q
Covariance matrix (statistic)	$\boldsymbol{\Sigma}_P$	$\boldsymbol{\Sigma}_Q$
Eigenvectors of covariance matrix	$\boldsymbol{P},\ P_k$	$\boldsymbol{Q},\ \ Q_k$
Eigenvalues of covariance matrix	λ_k^P	λ_k^Q
Diagonal matrix of eigenvalues	$\boldsymbol{\Lambda}_P$	$\boldsymbol{\Lambda}_Q$
Number of positive eigenvalues	pc_P	pc_Q
Number of eigenvalues retained	c_P	c_Q
‘Curated’ eigenvalues	$\hat{\lambda}_k^P$	$\hat{\lambda}_k^Q$

Supplementary Table 1: Symbolism used in mathematical grounds of the proposed dataset similarity method.

2.2 <u>The 'KL-divergence' of two standardized, multivariate normal distributions.</u>

Suppose two datasets $\boldsymbol{DS_P}[1..s_P, 1..n]$ and $\boldsymbol{DS_Q}[1..s_Q, 1..n]$ measuring the same number of variables *n*. Recall that a multivariate normal distribution $N(\boldsymbol{m}, \boldsymbol{\Sigma})$ with n variables, is defined by a pair $\langle \boldsymbol{m}, \boldsymbol{\Sigma} \rangle$ of a positive semi-definite matrix Σ (the 'covariance matrix'), along with a vector $\boldsymbol{m} = \langle m_1, m_2, \ldots, m_n, \rangle$ (the 'mean values', or the 'center'). For a positive definite matrix, the density can be defined as:

$$N(m, \Sigma)|_x \overset{def}{=} \frac{1}{\sqrt{(2\pi)^n |\Sigma|}} exp\left(-\frac{1}{2}(x-m)^T \Sigma^{(-1)}(x-m)\right) \qquad (2)$$

The KL-divergence between two normal distributions $N(\boldsymbol{m_P}, \boldsymbol{\Sigma}_P)$ and $N(\boldsymbol{m_q}, \boldsymbol{\Sigma}_Q)$ can be proven to be [4]:

$$KL(N(m_P, \Sigma_P)||N(m_P, \Sigma_P)) = \frac{1}{2}\left[trace\left(\Sigma_Q^{(-1)}\Sigma_P\right) + \left(m_Q - m_P\right)^T \Sigma_Q^{(-1)}\left(m_Q - m_P\right) + ln\left(\frac{|\Sigma_Q|}{|\Sigma_P|}\right) - n\right]$$

where $|\boldsymbol{\Sigma}| = det(\boldsymbol{\Sigma})$. For standardized normal distributions $\boldsymbol{m_P} = \boldsymbol{m_Q} = \boldsymbol{0}$ and the KL depends only on the covariance matrices and so

$$\boldsymbol{D(\Sigma_P||\Sigma_Q) \overset{def}{=} KL(N(0, \Sigma_P)||N(0, \Sigma_P)) = \frac{1}{2}\left[trace\left(\Sigma_Q^{(-1)}\Sigma_P\right) + ln\left(\frac{|\Sigma_Q|}{|\Sigma_P|}\right) - n\right]} \qquad (3)$$

Thus, under the assumption of standardized, multivariate normality, the KL depends only on the covariance matrices.

Geometrically, the contours of equal probability density *c* of a distribution *N(**0**, Σ)* are *ellipsoids* centered at ***0***. Moreover, the directions of the axes of these ellipsoids are given by the eigenvectors of $\boldsymbol{\Sigma}$, and the lengths of these axes are proportional to the square roots of the eigenvalues of $\boldsymbol{\Sigma}$, which coincide with the singular values of DS_P and DP_Q. Geometrically, $D(\Sigma_P||\Sigma_Q)$ computes a similarity between two high-dimensional, zero-centered ellipsoids corresponding to the same density with respect to their orientation and length of their axes. Numerically, $D(\Sigma_P||\Sigma_Q)$ compares the two matrices element-wise as it is proven (Lemma 2) that under a mild condition, $D(\boldsymbol{\Sigma}_P||\boldsymbol{\Sigma}_Q)$ is zero if only if the two matrices are equal. (Naturally, by appealing to standard calculus arguments about continuity and smoothness, we can infer that if $D(\boldsymbol{\Sigma}_P||\boldsymbol{\Sigma}_Q)$ takes 'small' values, then the difference of the two covariance matrices will also be 'small' in an also entry-wise manner). Our next goal is to derive a useful basic formula for $D(\boldsymbol{\Sigma}_P||\boldsymbol{\Sigma}_Q)$.

Lemma 1: Let $\boldsymbol{\Sigma}_P$ and $\boldsymbol{\Sigma}_Q$ be two positive definite covariance matrices of dimension *n*, with eigenvectors and eigenvalues as defined in Supplementary Supplementary Table 1. Their divergence $D(\boldsymbol{\Sigma}_P||\boldsymbol{\Sigma}_Q)$ is given by:

$$\boxed{D(\Sigma_P||\Sigma_Q) = \frac{1}{2}\left[\sum_{i=1}^{n}\sum_{j=1}^{n}\frac{\lambda_i^P}{\lambda_j^Q}\left(P_i{}^T \cdot Q_j\right)^2 + ln\left(\frac{\prod_{k=1}^{n}\lambda_k^Q}{\prod_{k=1}^{n}\lambda_k^P}\right) - n\right]} \qquad (4)$$

Proof: Any positive definite matrix $\boldsymbol{\Sigma}$ has a set of positive eigenvalues $\boldsymbol{\Lambda} = diag\langle \lambda_1, \lambda_2, \ldots, \lambda_n, \rangle$ and a matrix $\boldsymbol{V}$of n orthogonal eigenvectors, (= the columns V_k of $\boldsymbol{V}$), and so it can be written as:

$$\Sigma = V\Lambda V^T \tag{5}$$

Accordingly, the two positive definite matrices $\boldsymbol{\Sigma}_P$ and $\boldsymbol{\Sigma}_Q$ (arising from datasets 'P' and 'Q'), can be written as,

$$\Sigma_P = P\Lambda_P P^T,\ \Sigma_Q = Q\Lambda_Q Q^T \tag{6}$$

It is also known that,

$$|\Sigma_P| = det(\Sigma_P) = \prod_{k=1}^{n} \lambda_k^P \quad , \qquad |\Sigma_Q| = det(\Sigma_Q) = \prod_{k=1}^{n} \lambda_k^Q \tag{7}$$

Plugging the expressions (6) into the $trace(-)$ expression, we get:

$$trace\left(\Sigma_Q^{(-1)}\Sigma_P\right) = trace\left(\left(Q\Lambda_Q Q^T\right)^{(-1)}(P\Lambda_P P^T)\right) = trace\left(Q\Lambda_Q^{(-1)}Q^T P\Lambda_P P^T\right) \tag{8}$$

where, in the above derivation, we have exploited the fact that $\boldsymbol{Q}^{(-1)} = \boldsymbol{Q}^T$ (orthonormality of eigenvectors). Using the property $trace(\boldsymbol{X}\ (\boldsymbol{YZ})) = trace((\boldsymbol{YZ})\ \boldsymbol{X})$), we get,

$$trace\left(\Sigma_Q^{(-1)}\Sigma_P\right) = trace\left(Q\Lambda_Q^{(-1)}Q^T P\Lambda_P P^T\right) = trace\left(\Lambda_Q^{(-1)}(P^TQ)^T\Lambda_P(P^TQ)\right) \tag{9}$$

The matrix $\boldsymbol{\Lambda}_Q^{(-1)}$ is a diagonal matrix consisting of the inverses of the eigenvalues λ_k^Q, and the matrix $(\boldsymbol{P}^T\boldsymbol{Q})$ consists of the inner products $R_{\alpha,\beta} = \left(P_\alpha{}^T \cdot Q_\beta\right)$ of the (column) eigenvectors of 'P' and 'Q', over all pairs. We use the notation $[c(i,j)]_{i,j=1}^{n}$ to denote a square matrix $n \times n$ with element $c(i,j)$ on the ith row and j^{th} column and get:

$$\begin{aligned} trace\left(\Sigma_Q^{(-1)}\Sigma_P\right) &= trace\left(\left[\frac{1}{\lambda_\alpha^Q}R_{\beta,\alpha}\right]_{\alpha,\beta=1}^{n} \cdot \left[\lambda_\alpha^P R_{\alpha,\beta}\right]_{\alpha,\beta=1}^{n}\right) \\ &= trace\left(\left[\sum_{j=1}^{n}\frac{1}{\lambda_a^Q}R_{j,a}\ \ \lambda_j^P R_{j,\beta}\right]_{a,\beta=1}^{n}\right) \\ &= \textstyle\sum_{d=1}^{n}\sum_{j=1}^{n}\frac{\lambda_j^P}{\lambda_i^Q}\left(R_{j,d}\right)^2 = \sum_{i=1}^{n}\sum_{j=1}^{n}\frac{\lambda_i^P}{\lambda_j^Q}\left(P_i{}^T \cdot Q_j\right)^2 \end{aligned} \tag{10}$$

Summing up, we derive the stated formula (4).

□

We now explain what can be inferred when $D\left(\boldsymbol{\Sigma}_P || \boldsymbol{\Sigma}_Q\right) = 0$, in the following lemma:

Lemma 2: Let $\boldsymbol{\Sigma}_P$ and $\boldsymbol{\Sigma}_Q$ be two positive definite covariance matrices of dimension *n*, with discrete spectrums. $D\left(\boldsymbol{\Sigma}_P || \boldsymbol{\Sigma}_Q\right) = 0$ if and only if $\boldsymbol{\Sigma}_P$ equals $\boldsymbol{\Sigma}_Q$ entry-wise.

Proof: Since the eigenvectors form an orthonormal base, we have $\sum_{i=1}^{n}\left(P_i^T \cdot Q_j\right)^2 = 1$, j=1,..,n, $\sum_{j=1}^{n}\left(P_i^T \cdot Q_j\right)^2 = 1$, i=1,...,n, hence expression (4) takes the form:

$$D(\Sigma_P||\Sigma_Q) = \frac{1}{2}\sum_{i=1}^{n}\sum_{j=1}^{n}\left(\frac{\lambda_i^P}{\lambda_j^Q} - ln\frac{\lambda_i^P}{\lambda_j^Q} - 1\right)\left(P_i^T \cdot Q_j\right)^2 \tag{11}$$

Setting $x_{i,j} \leftarrow \left(P_i^T \cdot Q_j\right)^2$ and $\rho_{i,j} \leftarrow \left(\lambda_i^P/\lambda_j^Q\right)$, we get $x_{i,j} \geq 0,\ \sum_{i=1}^{n} x_{i,j} = 1$ and $\sum_{j=1}^{n} x_{i,j} = 1$. Thus, the possible values of $\left[(P_i^T \cdot Q_j)^2\right]_{i,j=1}^{n}$ are just a subset of the polytope **Π** of the doubly stochastic matrices $n \times n$, and expression (11) becomes the following linear function $L(\boldsymbol{x})$ over **Π**:

$$L(x) \overset{def}{=} \sum_{i=1}^{n}\sum_{j=1}^{n}(\rho_{i,j} - ln\,\rho_{i,j} - 1)x_{i,j} \tag{12}$$

Using the basic inequality $(\rho - 1) \geq ln\,\rho$ for $\rho > 0$, which is exact only in the case $\rho = 1$, we see that all terms in (12) are non-negative, hence the value 0 is the *minimum* possible value of (12), and hence also of (11). The minimum value of any linear function over a polytope is attained on a vertex. The vertices of **Π** are the permutation matrices $[x_{i,j}] = [\delta_{i,\ \alpha[j]}]$, where $\alpha[-]$is a permutation of $\{\ 1\ ..\ n\ \}$, and $\delta_{\kappa,\ \lambda}$ is the *Kronecker's delta.* On such a vertex, we get:

$$\boldsymbol{x_{i,\ \alpha[j]}} = \left(\boldsymbol{P_i^T \cdot Q_{\alpha[j]}}\right)^2 = \boldsymbol{\delta_{i,\ \alpha[j]}},\ \boldsymbol{L(x)} = \textstyle\sum_{k=1}^{n}\left(\boldsymbol{\rho_{k,\ \alpha[k]}} - \boldsymbol{ln}\left(\boldsymbol{\rho_{k,\ \alpha[k]}}\right) - \boldsymbol{1}\right) = \boldsymbol{0} \tag{13}$$

hence all the coefficients $\left(\rho_{k,\ \alpha[k]} - ln\left(\rho_{k,\ \alpha[k]}\right) - 1\right) \geq 0$ must be zero, or $\rho_{k,\ \alpha[k]} = 1 = \lambda_k^P/\lambda_{\alpha[k]}^Q,\quad k = 1,\ \ldots,\ n$. Therefore, the eigenvectors must be equal: $P_k = \pm Q_{\alpha[k]}$, $k = 1,\ldots,n$ (modulo a ± sign), and the eigenvalues must also be equal: $\lambda_k^P = \lambda_{\alpha[k]}^Q,\ k = 1,\ldots,n$ (one-to-one, under the same permutation).

This fact alone would complete our task in the case this minimizing vertex **x** was unique, since $\boldsymbol{\Sigma_P} = \boldsymbol{P\Lambda_P P^T}$, $\boldsymbol{\Sigma_Q} = \boldsymbol{Q\Lambda_Q Q^T}$ and the above matrix products do not depend on the numbering of the eigenvectors involved.

This is indeed the case, when the spectrums are discrete: for if $L(-)$were zero at a vertex $\boldsymbol{x'} \neq \boldsymbol{x}$ defined by permutation $\beta[-]$, where $\beta[i] \neq \alpha[i]$ for some i, by the above discussion we would get both $\lambda_i^P = \lambda_{\alpha[i]}^Q$ and $\lambda_i^P = \lambda_{\beta[i]}^Q$, or $\lambda_{\alpha[i]}^Q = \lambda_{\beta\ [i]}^Q$, contrary to our hypothesis of discrete spectrums.

It is easy to prove the reverse, i.e., that when $\boldsymbol{\Sigma_P} = \boldsymbol{\Sigma_Q}$, then $D\left(\boldsymbol{\Sigma_P}||\boldsymbol{\Sigma_Q}\right) = 0$. In this case, $trace\left(\Sigma_Q^{(-1)}\Sigma_P\right) = trace\left(\Sigma_P^{(-1)}\Sigma_P\right) = trace(I) = n$. By Eq. (7), $D\left(\boldsymbol{\Sigma_P}||\boldsymbol{\Sigma_Q}\right) = \frac{1}{2}(n - \ln 1 - n) = 0$.

□

If for two datasets $\boldsymbol{DS_P}[1..s_P, 1..n]$ and $\boldsymbol{DS_Q}[1..s_Q, 1..n]$, with sample covariance matrices $\boldsymbol{\Sigma_P}$ and $\boldsymbol{\Sigma_Q}$, we get relatively small values for the quantity $D\left(\boldsymbol{\Sigma_P}||\boldsymbol{\Sigma_Q}\right)$, we may deduce, by **Lemma 2**, that they are relatively similar *component-wise*. This means that the covariance of any pair of variables in 'P' is about the same as the covariance of the same pair of variables in 'Q'.

2.3 The 'curation' procedure for degenerate distributions.

In omics datasets the number of variables of datasets $\mathbf{DS}_P$, $\mathbf{DS}_Q$ is relatively large ($n > 50{,}000$), while the number of samples s is much smaller (e.g. $s < 100$). This makes the sample covariance matrices to be rank deficient, with most eigenvalues equal to zero; this fact makes formula (4) inapplicable (due to divisions by zero).

In order to make (4) applicable in the aforementioned cases, we will process ('curate') the spectrum of the eigenvalues, by adding the term $+\sigma$ to all of them.

To prepare a detailed presentation of this procedure, we mention two rather standard facts, for the manipulation of the covariance matrices and their respective eigenvalues:

Lemma 3: If we add to a covariance matrix a positive multiple of the identity matrix, setting $\boldsymbol{\Sigma} \leftarrow \boldsymbol{\Sigma} + \delta\ \boldsymbol{I},\ \ \delta > 0$, then the new matrix has the same set of eigenvectors V_k, and all its eigenvalues λ_k are increased by $+\delta$, (thus it remains a 'covariance matrix' and becomes positive definite, invertible, and full rank).

Proof: It suffices to calculate $(\boldsymbol{\Sigma} + \delta\ \boldsymbol{I})V_k$ which gives, $(\boldsymbol{\Sigma} + \delta \boldsymbol{I})V_k = \boldsymbol{\Sigma} V_k + \delta \boldsymbol{I} V_k = \lambda_k V_k + \delta V_k = (\lambda_k + \delta)V_k$

□

Lemma 4: The inverse $\boldsymbol{\Sigma}^{(-1)}$, of a covariance matrix $\boldsymbol{\Sigma}$, (when it exists), has the same set of eigenvectors V_k, and its eigenvalues are the inverses of the original eigenvalues λ_k.

Proof: Since $\boldsymbol{\Sigma}\ V_k = \lambda_k V_k$, multiplying by $\boldsymbol{\Sigma}^{(-1)}$ gives $\boldsymbol{\Sigma}^{(-1)}(\lambda_k V_k) = \boldsymbol{\Sigma}^{(-1)}\ (\boldsymbol{\Sigma}\ V_k) = V_k$, thus: $\boldsymbol{\Sigma}^{(-1)}\ V_k = \frac{1}{\lambda_k} V_k$

□

The $\langle \alpha, \sigma \rangle$ 'curation' procedure of the covariance matrix:

Given a covariance matrix $\boldsymbol{\Sigma}$ of a dataset **DS**, with a number pc of positive eigenvalues λ_k, (typically substantially less than n), we proceed to the following 'curation' of $\boldsymbol{\Sigma}$, depending on two parameters α and σ, where $0 < \alpha \leq 1$, and $\sigma > 0$. The procedure filters only the most important eigenvectors. Notice below, that due to normalization of the variables of the datasets, the matrix $\boldsymbol{\Sigma}$ has unit elements along its diagonal as already mentioned, thus,

$$\sum_{k=1}^{n} \lambda_k = trace(\Sigma) = \sum_{k=1}^{n} 1 = n \tag{14}$$

Next, we consider the eigenvalues indexed in descending order, i.e., $\lambda_1 > \lambda_2 > \ldots > \lambda_s$. It is also reasonable to assume that in most typical cases with high-dimensional data and small sample sizes the eigenvalues will not be equal (discrete spectrum). We only maintain the first c eigenvalues and corresponding eigenvectors. The value of c depends on a:

Step 1 (*filtration*): select an index c, $1 \leq c \leq pc$ such that,

$$c \ = argmin_{\{c' \in N\}} \left(\sum_{i=1}^{c'} \lambda_i \ \ \geq \alpha \ \ n \right)$$

and set

$$\lambda_k^{'} \leftarrow \lambda_k, \quad k = 1, \ldots, c$$
$$\lambda_k^{'} \leftarrow 0, \quad k = c + 1, \ldots, n$$

Justification: due to the small sample size, we consider the estimation of the eigenvectors corresponding to the smaller eigenvalues unreliable. Thus, only the first *c* eigenvalues are retained, and the rest are set to 0. The 'tuning' of the value of parameter α is commented in the main text. The parameter *a* can be interpreted as the smallest percentage of variance to retain with the selected eigenvectors.

Step 2 (*scaling*): Set $l = \sum_{k=1}^{c} \lambda_k$, scale by *αn*/*l* the first c largest eigenvalues:

$$\lambda_k^{'} \leftarrow \lambda_k^{'} \left(\frac{\alpha n}{l}\right), \quad k = 1, \ldots, c$$

Justification: we scale the first *c* eigenvalues, by a factor $\frac{\alpha n}{l}$ so that the scaled eigenvalues add exactly to *an*: $\alpha n = \sum_{k=1}^{c} \lambda'_k$. Subsequent experimentation showed that this step improves comparison of KLs computed between different pairs of datasets.

*Step*3 (*inflation*): Add a constant $\sigma > 0$ to all the *n* eigenvalues:

$$\lambda_k^{''} \leftarrow \lambda_k^{'} + \sigma, \quad k = 1, \ldots, n$$

Justification: essentially, this is equivalent to adding a 'noise' term ν_k to each variable $x_k \leftarrow x_k + \nu_k, k = 1, \ldots, n$, with zero mean, variance equal to σ, and zero covariance with (uncorrelated to) all other variables. In other words, we add a noise term of equal variance to each direction in the data space, which is why this term is called an isotropic noise component. Notice that this is equivalent to changing $\boldsymbol{\Sigma}$ to $\boldsymbol{\Sigma} + \sigma\boldsymbol{I}$, a fact that increases all the eigenvalues by $+\sigma$ (see **Lemma 3**).

In summary, the ⟨α,σ⟩ curated eigenvalues are:

$$c = argmin_{\{c' \in N\}} \left(\sum_{i=1}^{c'} \lambda_i \geq \alpha \ n \right),$$

$$\hat{\lambda}_k = \frac{\alpha n}{\sum_{k=1..n} \lambda_k} \lambda_k + \sigma, \quad k = 1, \ldots, c,$$

$$\hat{\lambda}_k = \sigma, \qquad k = c + 1, \ldots, n \tag{15}$$

The procedure leaves the eigenvectors intact. All eigenvalues are now positive, hence formula (4) is applicable.

Notice that **by setting** $\boldsymbol{\sigma} \leftarrow (\mathbf{1} - \boldsymbol{\alpha})$ we readjust the total variance back to the level *n* (see (14)): $\sum_{k=1}^{n} \hat{\lambda}_k = \sum_{k=1}^{c} (\lambda'_k + \sigma) + (n - c)\sigma = \alpha n + c\sigma + n\sigma - c\sigma = \alpha n + n(1 - \alpha) = n$. Notice also, that with respect to the ellipsoidal visualization of $\boldsymbol{\Sigma}_P$ and $\boldsymbol{\Sigma}_Q$, the above curation procedure essentially 'inflates' the ellipsoids defined by $\boldsymbol{\Sigma}_P$ and $\boldsymbol{\Sigma}_Q$, extending every axis of them by a $+\sigma$ term. In typical omics datasets the largest eigenvalues are in the order of thousands, while σ is in the order of 0.5, thus the procedure has a negligible effect on the top eigenvalues.

Obviously, the same 'curation' technique is applied to both covariance matrices $\boldsymbol{\Sigma}_P$, $\boldsymbol{\Sigma}_Q$, determining thus the number of retained eigenvalues $c_P \leftarrow c$ and $c_Q \leftarrow c$, and their curated values, $\hat{\lambda}_k^P \leftarrow \hat{\lambda}_k, \hat{\lambda}_k^Q \leftarrow \hat{\lambda}_k$. Typical choices for α and σ are $\alpha = \alpha_P = \alpha_Q = 50\%$ and $\sigma = \sigma_P = \sigma_Q = 0.5$.

In the curated spectrum all the eigenvalues, except the few largest ones, will have a small *constant* value (σ_P or σ_Q), and due to this fact, formula (4) is not only applicable but greatly simplified.

Lemma 5: Let $\boldsymbol{\Sigma}_P$ and $\boldsymbol{\Sigma}_Q$ be two covariance matrices of dimension *n*, with eigenvectors P_k, Q_k and eigenvalues λ_k^P, λ_k^Q, respectively. Furthermore, let the last $n - c_P$ eigenvalues of $\boldsymbol{\Sigma}_P$ be equal to σ_P, and the last $n - c_Q$ eigenvalues of $\boldsymbol{\Sigma}_Q$ be equal to σ_Q. The divergence $D\left(\boldsymbol{\Sigma}_P || \boldsymbol{\Sigma}_Q\right)$ is given by:

$$D\left(\Sigma_P || \Sigma_Q\right) = \frac{1}{2}\frac{\sigma_P}{\sigma_Q}\left[n - \left(c_P + c_Q\right) + \left(\sum_{k=1}^{c_P}\frac{\lambda_k^P}{\sigma_P} + \sum_{k=1}^{c_Q}\frac{\sigma_Q}{\lambda_k^Q}\right) - \sum_{i=1}^{c_P}\sum_{j=1}^{c_Q}\left(\frac{\lambda_i^P}{\sigma_P} - 1\right)\left(1 - \frac{\sigma_Q}{\lambda_j^Q}\right)\left({P_i}^T \cdot Q_j\right)^2\right] + \frac{1}{2}\left[ln\left(\frac{\prod_{k=1}^{c_Q}\lambda_k^Q}{\prod_{k=1}^{c_P}\lambda_k^P}\frac{\sigma_Q^{(n-c_Q)}}{\sigma_P^{(n-c_P)}}\right) - n\right] \quad (16)$$

Proof: We use the general formula (4) of the *KL*-divergence obtained previously, and focus on the $trace(-)$ term, which we split it into four parts:

$$trace\left(\Sigma_Q^{(-1)}\Sigma_P\right) = \sum_{i=1}^{n}\sum_{j=1}^{n}\frac{\lambda_i^P}{\lambda_j^Q}\left({P_i}^T \cdot Q_j\right)^2$$

$$\begin{aligned}
&= \sum_{i=1}^{c_P}\sum_{j=1}^{c_Q}\frac{\lambda_i^P}{\lambda_j^Q}\left({P_i}^T \cdot Q_j\right)^2 && 1^{st}\ term \\
&+ \sum_{i=1}^{c_P}\sum_{j=c_Q+1}^{n}\frac{\lambda_i^P}{\sigma_Q}\left({P_i}^T \cdot Q_j\right)^2 && 2^{nd}\ term \\
&+ \sum_{i=c_P+1}^{n}\sum_{j=1}^{c_Q}\frac{\sigma_P}{\lambda_j^Q}\left({P_i}^T \cdot Q_j\right)^2 && 3^{rd}\ term \\
&+ \sum_{i=c_P+1}^{n}\sum_{j=c_P+1}^{n}\frac{\sigma_P}{\sigma_Q}\left({P_i}^T \cdot Q_j\right)^2 && 4^{th}\ term
\end{aligned} \quad (17)$$

Since the eigenvectors are normalized (that is: they have unit Euclidean norm), and they form an orthonormal base of the whole space, we get $\sum_{i=1}^{n}\left({P_i}^T \cdot Q_j\right)^2 = 1$ for every j, hence,

$$\sum_{i=1}^{n}\sum_{j=1}^{n}\left({P_i}^T \cdot Q_j\right)^2 = n,$$

$$\sum_{i=c_P+1}^{n}\left({P_i}^T \cdot Q_j\right)^2 = 1 - \sum_{i=1}^{c_P}\left({P_i}^T \cdot Q_j\right)^2, \ \sum_{j=c_Q+1}^{n}\left({P_i}^T \cdot Q_j\right)^2 = 1 - \sum_{j=1}^{c_Q}\left({P_i}^T \cdot Q_j\right)^2 \quad (18)$$

Using expressions (18) in the 2nd and 3rd terms of expression (17) for the $trace\left(\boldsymbol{\Sigma}_Q^{(-1)}\boldsymbol{\Sigma}_P\right)$ we have:

$$\sum_{i=1}^{c_P}\sum_{j=c_Q+1}^{n}\frac{\lambda_i^P}{\sigma_Q}\left({P_i}^T \cdot Q_j\right)^2 = \frac{\sigma_P}{\sigma_Q}\sum_{i=1}^{c_P}\frac{\lambda_i^P}{\sigma_P}\left(1 - \sum_{i=1}^{c_Q}\left({P_i}^T \cdot Q_j\right)^2\right),$$

$$\sum_{i=c_P+1}^{n}\sum_{j=1}^{c_Q}\frac{\sigma_P}{\lambda_j^Q}\left({P_i}^T \cdot Q_j\right)^2 = \frac{\sigma_P}{\sigma_Q}\sum_{j=1}^{c_Q}\frac{\sigma_Q}{\lambda_j^Q}\left(1 - \sum_{i=1}^{c_P}\left({P_i}^T \cdot Q_j\right)^2\right) \quad (19)$$

For the 4th term of (17) we have:

$$\sum_{i=c_P+1}^{n}\sum_{j=c_P+1}^{n}\left(P_i^{\ T}\cdot Q_j\right)^2$$
$$=\sum_{i=1}^{n}\sum_{j=1}^{n}\left(P_i^{\ T}\cdot Q_j\right)^2-\sum_{i=1}^{c_P}\sum_{j=c_Q+1}^{n}\left(P_i^{\ T}\cdot Q_j\right)^2-\sum_{i=c_P+1}^{n}\sum_{j=1}^{c_Q}\left(P_i^{\ T}\cdot Q_j\right)^2-\sum_{i=1}^{c_P}\sum_{j=1}^{c_Q}\left(P_i^{\ T}\cdot Q_j\right)^2 \qquad (20)$$

Using the identities (18) in the previous formula (20) we get:

$$\sum_{i=c_P+1}^{n}\sum_{j=c_P+1}^{n}\left(P_i^{\ T}\cdot Q_j\right)^2$$
$$=n-\sum_{i=1}^{c_P}\left(1-\sum_{j=1}^{c_Q}\left(P_i^{\ T}\cdot Q_j\right)^2\right)-\sum_{j=1}^{c_Q}\left(1-\sum_{i=1}^{c_P}\left(P_i^{\ T}\cdot Q_j\right)^2\right)-\sum_{i=c_P+1}^{n}\sum_{j=c_P+1}^{n}\left(P_i^{\ T}\cdot Q_j\right)^2 \qquad (21)$$

or finally, for the 4th term in (17):

$$\frac{\sigma_P}{\sigma_Q}\sum_{i=c_P+1}^{n}\sum_{j=c_P+1}^{n}\left(P_i^{\ T}\cdot Q_j\right)^2=\frac{\sigma_P}{\sigma_Q}\left[\left(n-c_P-c_Q\right)+\sum_{i=1}^{c_P}\sum_{j=1}^{c_Q}\left(P_i^{\ T}\cdot Q_j\right)^2\right] \qquad (22)$$

Adding all four terms in (17) together, dragging the ratio $\frac{\sigma_P}{\sigma_Q}$ outside the whole expression, and using the simple fact,

$$\left(\frac{\boldsymbol{\lambda_i^P}}{\boldsymbol{\sigma_P}}\right)\left(\frac{\boldsymbol{\sigma_Q}}{\boldsymbol{\lambda_i^Q}}\right)-\left(\frac{\boldsymbol{\lambda_i^P}}{\boldsymbol{\sigma_P}}\right)-\left(\frac{\boldsymbol{\sigma_Q}}{\boldsymbol{\lambda_i^Q}}\right)+\mathbf{1}=\left(\frac{\boldsymbol{\lambda_i^P}}{\boldsymbol{\sigma_P}}-\mathbf{1}\right)\left(\frac{\boldsymbol{\sigma_Q}}{\boldsymbol{\lambda_i^Q}}-\mathbf{1}\right) \qquad (23)$$

we get,

$$trace\left(\Sigma_Q^{(-1)}\Sigma_P\right)=$$
$$\frac{\sigma_P}{\sigma_Q}\left[\left(n-c_P-c_Q\right)+\left(\sum_{k=1}^{c_P}\frac{\lambda_k^P}{\sigma_P}+\sum_{k=1}^{c_Q}\frac{\sigma_Q}{\lambda_k^Q}\right)-\sum_{i=1}^{c_P}\sum_{j=1}^{c_Q}\left(\frac{\lambda_i^P}{\sigma_P}-1\right)\left(1-\frac{\sigma_Q}{\lambda_j^Q}\right)\left(P_i^{\ T}\cdot Q_j\right)^2\right] \qquad (24)$$

Substituting the above expression to the basic formula (4), we get what is stated in formula (16).

□

Expression (16) is not 'simple' in a visual manner, but it is simpler from a computational point of view: for $n \approx 50{,}000$ and low values of c_P, c_Q, the computation of (16) consumes several thousand times less memory, and million times less computational power than formula (4).

2.4 Symmetric divergence: approximation and robustness.

Since the *KL*-divergence is not a symmetric function, in the main text we use a symmetrical variation of it, defined as follows:

$$\boldsymbol{SKL}\left(\boldsymbol{\Sigma_P},\boldsymbol{\Sigma_Q}\right)\ \stackrel{\boldsymbol{def}}{=}\ \left[\boldsymbol{D}\left(\boldsymbol{\Sigma_P}||\boldsymbol{\Sigma_Q}\right)+\boldsymbol{D}\left(\boldsymbol{\Sigma_Q}||\boldsymbol{\Sigma_P}\right)\right] \qquad (25)$$

The following simple Lemma gives the 'simplified' formula for this version of the *KL*-divergence:

Lemma 6 (*symmetric divergence***)**: Let $\boldsymbol{\Sigma}_P$, $\boldsymbol{\Sigma}_Q$ be the (statistical) covariance matrices of two datasets 'P' and 'Q'. Let $\widehat{\boldsymbol{\Sigma}}_P$, $\widehat{\boldsymbol{\Sigma}}_Q$ be the 'curated' covariance matrices, resulting from the curation procedure given in (15), with parameters $\langle\alpha,\ \sigma\rangle$ for both datasets. The value of $c\text{-}SKL\left(\widehat{\boldsymbol{\Sigma}}_P,\widehat{\boldsymbol{\Sigma}}_Q\right)$ (*c* stands for curated) is given by the following expression:

$$c\text{-}SKL\left(\hat{\Sigma}_P,\hat{\Sigma}_Q\right)=\frac{1}{2}\left[\sum_{k=1}^{c_P}\left(\frac{\hat{\lambda}_k^P}{\sigma}+\frac{\sigma}{\hat{\lambda}_k^P}\right)+\sum_{k=1}^{c_Q}\left(\frac{\hat{\lambda}_k^Q}{\sigma}+\frac{\sigma}{\hat{\lambda}_k^Q}\right)-2\left(c_P+c_Q\right)\right]+\frac{1}{2}\left[-\sum_{i=1}^{c_P}\sum_{j=1}^{c_Q}\left(\frac{\hat{\lambda}_i^P}{\sigma}-1\right)\left(1-\frac{\sigma}{\hat{\lambda}_j^Q}\right)\left(P_i^{\ T}\cdot Q_j\right)^2-\right.$$
$$\left.\sum_{i=1}^{c_P}\sum_{j=1}^{c_Q}\left(1-\frac{\sigma}{\hat{\lambda}_i^P}\right)\left(\frac{\hat{\lambda}_j^Q}{\sigma}-1\right)\left(P_i^{\ T}\cdot Q_j\right)^2\right] \qquad (26)$$

Proof: Notice that the curated eigenvalues $\hat{\lambda}_k^P$, $\hat{\lambda}_k^Q$ after the first c_P, $(resp.\ \ c_Q)$ ones, are equal to $\sigma_P = \sigma_Q = \sigma$. The above formula is derived by substituting the 'curated' eigenvalues of $\boldsymbol{\Sigma}_P$, $\boldsymbol{\Sigma}_Q$ to (16) and taking the sum, $D(\widehat{\boldsymbol{\Sigma}}_P||\widehat{\boldsymbol{\Sigma}}_Q) + D(\widehat{\boldsymbol{\Sigma}}_Q||\widehat{\boldsymbol{\Sigma}}_P)$. Notice that the two logarithmic terms of (16) containing the products of the eigenvalues, cancel each other.

□

Finally, we give an approximate formula valid for the cases we handle in the main text. It has a simple form that allows us to develop an insight on what determines the value of the *SKL* divergence.

Lemma 7 (*approximate SKL***)**: If the number of the retained eigenvalues is small ($c_P,\ \ c_Q < < n$); their values are large enough ($\lambda_k^P,\ \lambda_k^Q >> 1$); and the '$\sigma$' used in the curation procedure is small compared to the retained eigenvalues, then the symmetric divergence $SKL(\widehat{\boldsymbol{\Sigma}}_P, \widehat{\boldsymbol{\Sigma}}_Q)$ is approximated by $\overline{SKL}_{\langle\alpha,\sigma\rangle}(\boldsymbol{\Sigma}_P, \boldsymbol{\Sigma}_Q)$ defined as follows:

$$c\text{-}\overline{SKL}_{\langle\alpha,\sigma\rangle}(\Sigma_P,\Sigma_Q) \overset{def}{=} \frac{1}{2\sigma}\left[2\alpha n - \sum_{i=1}^{c_P}\sum_{j=1}^{c_Q}\lambda_i^P\left(P_i^T\cdot Q_j\right)^2 - \sum_{i=1}^{c_P}\sum_{j=1}^{c_Q}\lambda_j^Q\left(P_i^T\cdot Q_j\right)^2\right] \quad (27)$$

Proof: Since $\sum_{k=1}^{c_P}\hat{\lambda}_k^P = \alpha n + c_P\sigma$, (see 'curation' form (15)), we get $\sum_{k=1}^{c_P}\frac{\hat{\lambda}_k^P}{\sigma} = \frac{\alpha}{\sigma}n + c_P$. The sum $\sum_{k=1}^{c_P}\sigma/\hat{\lambda}_k^P$ has a negligible value. Each factor $(\hat{\lambda}_k^P/\sigma) - 1$ is essentially equal to $\frac{1}{\sigma}\lambda_k^P$, and the factors $1 - (\sigma/\hat{\lambda}_k^P)$ are essentially equal to 1. The same facts hold, correspondingly, for the terms containing the 'Q' eigenvalues. The terms c_P and $c_Q$are also negligible with respect to αn. Any remaining unneglectable quantities (25) are those contained in (27), thus, $SKL(\widehat{\boldsymbol{\Sigma}}_P, \widehat{\boldsymbol{\Sigma}}_Q) \approx \overline{SKL}_{\langle\alpha,\sigma\rangle}(\boldsymbol{\Sigma}_P, \boldsymbol{\Sigma}_Q)$.

□

The above formula is useful for two purposes. First, it offers a simple way of 'visualizing' the way the *KL*-divergence is computed, using the ellipsoids associated with $\boldsymbol{\Sigma}_P$ and $\boldsymbol{\Sigma}_Q$: In (27), we can see that the term $\sum_{j=1}^{c_Q}\lambda_i^P\left(P_i^T\cdot Q_j\right)^2$ equals (the square of) the length of the *projection* of the $\left\langle\sqrt{\lambda_i^P}, P_i\right\rangle$ axis of 'P'-ellipsoid, on the subspace of the 'Q'-ellipsoid. The whole formula adds up the two projections. Since this sum is subtracted from the term $2\alpha n$, the largest the projections, the smaller the divergence will be.

Second, formula (27) leads us to the following last, but also quite useful, Lemma:

Lemma 8 (*robustness of ranking***)**: Let $\widehat{\boldsymbol{\Sigma}}_P$,$\widehat{\boldsymbol{\Sigma}}_{Q1}$ and $\widehat{\boldsymbol{\Sigma}}_{Q2}$ be three curated covariance matrices such that, $\overline{SKL}_{\langle\alpha,\sigma\rangle}(\boldsymbol{\Sigma}_P, \boldsymbol{\Sigma}_{Q1}) \leq \overline{SKL}_{\langle\alpha,\sigma\rangle}(\boldsymbol{\Sigma}_P, \boldsymbol{\Sigma}_{Q2})$. If we set the parameter σ to a new value $\sigma*$ (again 'small' with respect to the eigenvalues retained), the above ordering will be preserved: $\overline{SKL}_{\langle\alpha,\sigma*\rangle}(\boldsymbol{\Sigma}_P, \boldsymbol{\Sigma}_{Q1}) \leq \overline{SKL}_{\langle\alpha,\sigma*\rangle}(\boldsymbol{\Sigma}_P, \boldsymbol{\Sigma}_{Q2})$.

Proof: This holds because both $\overline{SKL}_{\langle\alpha,\sigma\rangle}$ terms will only be scaled by the same factor $\sigma/\sigma*$.

□

The meaning of the above Lemma is that although the choice of curation parameter σ does affect in an arbitrary manner the *absolute* value of the symmetric *KL*-divergence, it does not affect the

relative ranking of the (approximate) ‘similarity’ between the datasets. Although the result refers to the approximate formula $\overline{SKL}_{\langle\alpha,\sigma\rangle}$, it is applied to our experimental results.

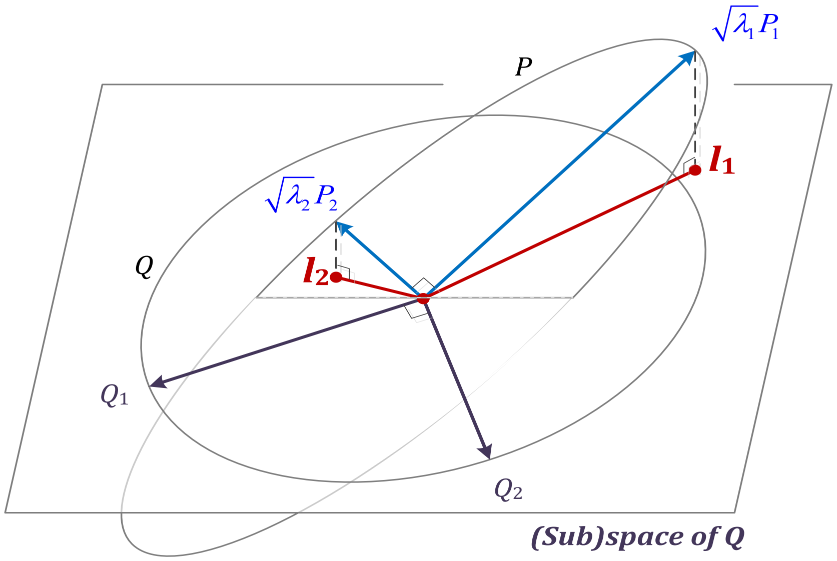

Supplementary Figure 3: A schematic representation of the KL-divergence between two statistical distributions. Distributions (depicted here by their PCA ellipsoids) lie on two different planes (hyperplanes in general). The resulting divergence depends approximately on how large (in total, red color) the eigenvectors of one dataset remain when projected onto the subspace of the other dataset.

2.4 Determining statistical significance of an edge

To estimate the statistical significance of an edge $E_{P,Q} = \{(\mathrm{v}_P, \mathrm{v}_Q): \mathrm{v}_P, \mathrm{v}_Q \in \mathrm{V}, \mathrm{v}_P \neq \mathrm{v}_Q\}$ between two nodes v_P and v_Q that represent datasets from two different statistical distributions, DS_P and DS_Q, we compute the $c\text{-}\overline{SKL}_{\langle\alpha,\sigma\rangle}(\Sigma_P, \Sigma_Q)$ between DS_P and 20 randomly formed datasets $\mathrm{DS}_{Q'}$ with the same sample size as DS_Q. All samples included in those datasets are drawn from the pooled joint distribution of all samples of each technology. For simplicity we denote the distribution of $c\text{-}\overline{SKL}_{\langle\alpha,\sigma\rangle}\left(\Sigma_P, \Sigma_{Q_i'}\right)$ where $i = 1{:}\,20,$ as $X_{P\to Q}$ and the distribution of $c\text{-}\overline{SKL}_{\langle\alpha,\sigma\rangle}\left(\Sigma_{P_i'}, \Sigma_Q\right)$ as $X_{Q\to P}$. We then fit a univariate normal distribution $F\left(X_{P\to Q}\right)\sim N(\mu_{X_{P\to Q}}, \sigma^2_{X_{P\to Q}})$ and $F\left(X_{Q\to P}\right)\sim N(\mu_{X_{Q\to P}}, \sigma^2_{X_{Q\to P}})$, by maximum likelihood estimation, to $X_{P\to Q}$ and $X_{Q\to P}$ respectively, and designate an edge $E_{P,Q}$ as statistically significant, if and only if, $c\text{-}\overline{SKL}_{\langle\alpha,\sigma\rangle}\left(\Sigma_P, \Sigma_Q\right) \leq F\left(X_{P\to Q}\right)_{0.025}$ & $\overline{SKL}_{\langle\alpha,\sigma\rangle}\left(\Sigma_Q, \Sigma_P\right) \leq F\left(X_{Q\to P}\right)_{0.025}$, where $F\left(X_{P\to Q}\right)_{0.025}$ is the lower 5% percentile of $X_{P\to Q}$ and $F\left(X_{Q\to P}\right)_{0.025}$ the lower 5% percentile of $X_{Q\to P}$, respectively.

To weight an edge according to its statistical significance, we compute how many standard deviations fit in the distance between the observed *c-SKL* and μ_X, as: $w_{E_{P,Q}} = \min\left\{\frac{\left|\overline{SKL}_{\langle\alpha,\sigma\rangle}(\Sigma_P,\Sigma_Q)-\mu_{X_{P\to Q}}\right|}{\sigma_{X_{P\to Q}}}, \frac{\left|\overline{SKL}_{\langle\alpha,\sigma\rangle}(\Sigma_P,\Sigma_Q)-\mu_{X_{Q\to P}}\right|}{\sigma_{X_{Q\to P}}}\right\}$. Intuitively, we weight each edge by its statistical significance compared to the pooled joint distribution from all measured samples.

2.5 Explaining SKL

Here we design a heuristic algorithm to identify the top *k* variables that are the most responsible for an observed *c-SKL*. Consider two datasets $\boldsymbol{DS_P}$ and $\boldsymbol{DS_Q}$ with covariance matrices decomposed as $\Sigma_P = P\Lambda^P P^T + \sigma_P I$ and $\Sigma_Q = Q\Lambda^Q P^Q + \sigma_Q I$ where *P* and *Q* are the matrices with the first c_P and c_Q principal components, Λ^{P} and Λ^{Q} the diagonal matrices with the corresponding eigenvalues $\lambda_i^P, i = 1, \dots, c_P$ and $\lambda_i^Q, i = 1, \dots, c_Q$ on the diagonal. After scaling the eigenvalues so $\sum_{i=1}^{c_P} \lambda_i^P = \sum_{i=1}^{c_Q} \lambda_i^Q = \alpha$, then $\sigma_P = \sigma_Q = \sigma$. The exact *c-SKL* divergence is given by (26). Let us call *c-SKL*(*S*), where $S_{n\times 1}$ is a vector of zeros and ones, the *c-SKL* divergence between D_P and D_Q when computed only on the variables with a corresponding one in *S*. We can now consider the following optimization problem:

$$B(k) = argmin_I\, SKL(S), \text{ s.t,. } S \text{ is a zero-one vector of size } n \text{, with sum equal to } k$$

The problem above selects exactly *k* variables that make the two datasets seem most similar, with respect to the *SKL*. Equivalently, the problem can be written as:

$$B(k) = argmax_I \sum_{i=1}^{c_P} \sum_{j=1}^{c_Q} C_{ij} \left(P_i^T diag(I) Q_j\right)^2 \qquad (28)$$

where *diag(I)* produces a diagonal matrix with *S* in its diagonal, and $C_{ij}^{PQ} = \left(\frac{\lambda_i^P}{\sigma} - 1\right)\left(1 - \frac{\sigma}{\lambda_j^Q}\right) + \left(\frac{\lambda_j^Q}{\sigma} - 1\right)\left(1 - \frac{\sigma}{\lambda_i^P}\right)$. The superscripts *P, Q* on *c* denote its dependence to the specific datasets with principal components in *P* and *Q* respectively. They are dropped when it is clear the pair *P* and *Q* they correspond to; we use the superscripts only later when we consider explaining all *c-SKL*s among a group of datasets.

In its bilinear form, the equation can be written as:

$$B(k) = argmax_{T,S} \sum_{i=1}^{c_P} \sum_{j=1}^{c_Q} C_{ij} \left(P_i^T diag(T) Q_j\right)\left(P_i^T diag(S) Q_j\right) \quad (29)$$

where *T* and *S* are again zero-one vectors of size *n*, with exactly *k* ones. Expanding, in order to get the equation to the standard quadratic form, we get:

$$B(k) = argmax_{T,S} \sum_{i=1}^{c_P} \sum_{j=1}^{c_Q} C_{ij} \left(\sum_{t=1}^{n} P_{ti} Q_{tj} T_t\right)\left(\sum_{s=1}^{n} P_{si} Q_{sj} S_s\right) =$$

$$argmax_{T,S} \sum_{t=1}^{n} \sum_{s=1}^{n} \sum_{i=1}^{c_P} \sum_{j=1}^{c_Q} C_{ij} P_{ti} Q_{tj} T_t P_{si} Q_{sj} S_s = \quad (30)$$

$$argmax_{T,S} \sum_{t=1}^{n} \sum_{s=1}^{n} T_t \left(\sum_{i=1}^{c_P} \sum_{j=1}^{c_Q} C_{ij} P_{ti} Q_{tj} P_{si} Q_{sj}\right) S_s = argmax_{T,S} T^T H S$$

where *H* is a matrix with elements $H_{ts} = \sum_{i=1}^{c_P} \sum_{j=1}^{c_Q} C_{ij} P_{ti} Q_{tj} P_{si} Q_{sj}$. Obviously, *H* is symmetric (the expression evaluates the same if we exchange indexes *s* and *t*). Thus, the bilinear theorem holds and so if the pair *T* and *S* with *T*=*S* is a solution to the bilinear problem, then *T*=*S* is a solution to the original problem [5]. Also, the guaranties of the simple algorithm where one starts with an initial guess for *T*, solves a linear program for *S*, and alternates until convergence can be found in [5]. This simple, heuristic algorithm is employed in our implementation. Notice that, matrix *H* is of size *n*×*n* and thus, impractical to construct for typical omics datasets where *n* ranges in the tens of thousands. It is presented here only to prove that *H* is symmetric and that the bilinear theorem can be applied. In practice, we solve the problem without explicitly constructing *H* as follows, starting from the equation above and considering *S* as known and fixed:

$$\sum_{i=1}^{c_P} \sum_{j=1}^{c_Q} C_{ij} \left(\sum_{t=1}^{n} P_{ti} Q_{tj} T_t\right)\left(\sum_{s=1}^{n} P_{si} Q_{sj} S_s\right) = \sum_{i=1}^{c_P} \sum_{j=1}^{c_Q} C_{ij} \left(\sum_{t=1}^{n} P_{ti} Q_{tj} T_t\right) W_{ij} \quad (31)$$

where $W = (P^T diag(S) Q) = \left(\sum_{s=1}^{n} P_{si} Q_{sj} S_s\right)$. Defining *Z* = *C* • *W*, where • denotes the Hadamard (element-wise) product of matrices, we obtain:

$$\sum_{i=1}^{c_P} \sum_{j=1}^{c_Q} Z_{ij} \left(\sum_{t=1}^{n} P_{ti} Q_{tj} T_t\right) = \sum_{t=1}^{n} T_t \sum_{i=1}^{c_P} \sum_{j=1}^{c_Q} Z_{ij} P_{ti} Q_{tj} = \sum_{t=1}^{n} T_t R_t \quad (32)$$

where $R = diag(PZQ^T)$, and *diag* returns the diagonal of its input. Matrix PZQ^T is an *n*-by-*n* matrix but of course, only its *n* diagonal elements need to be computed. (In fact, one could compute *R* using matrix multiplications only as [(*P*·*Z*) • *Q*] · **1**, where **1** denotes a column vector of ones). Overall, $R = diag(P \cdot (C \bullet (P^T \cdot \mathrm{diag}(S) \cdot Q) \cdot Q^T)$, where it can be seen that no intermediate results require the construction of any *n*-by-*n* matrices. Once *R* is computed it is trivial to find the *T* that maximizes $\sum_{t=1}^{n} T_t R_t$: we set to 1 the *k* elements of *T* that correspond to the largest values of *R*. This can be found with a simple sorting algorithm in *O(nlogn)* time. Similarly, we solve for *S* when *T* is hold fixed. We can also define the problem as:

$$W(k) = argmix_I \sum_{i=1}^{c_P} \sum_{j=1}^{c_Q} C_{ij} \left(P_i^T diag(S) Q_j\right)^2 \quad (33)$$

where *S* is again a zero-one vector with exactly *k* ones. *W(k)* correspond to the *k* variables in the two datasets that **mostly contribute to making their *c-SKL* divergence large**. The set of molec-

ular quantities (genes in our case) that correspond to *W(k)* can be employed to identify the pathways that are most different in the two datasets. Finally, one may be interested in the set of k gene expressions, methylations and variables in general, which best explain the similarity among **a group of datasets** with corresponding principal component matrices in the set $\Xi=\{P_1, \ldots, P_g\}$ and corresponding eigenvalues $\{\Lambda_1, \ldots, \Lambda_g\}$. For this scenario we define and solve the problem:

$$B(k) = argmax_I \sum_{P\neq Q\in\Xi} \sum_{i=1}^{c_P} \sum_{j=1}^{c_Q} C_{ij}^{PQ} \left(P_i^T diag(S) Q_j\right)^2 \quad (34)$$

where the selection of top explaining variables considers simultaneously all SKLs between every pair of datasets represented with its principal components and eigenvalue matrices. Notice that the coefficient matrix C now depends on the eigenvalues of the datasets compared and we employ the superscripts P and Q to denote this fact. The equation in its bilinear form, solving for T and considering S as fixed, is reduced to solving in each iteration:

$$argmax_T \sum_{t=1}^{n} T_t \sum_{P\neq Q\in\Xi} R_t^{PQ} \quad (35)$$

where $R^{PQ} = diag(P \cdot (C^{PQ} \bullet (P^T \cdot \mathrm{diag}(S) \cdot Q) \cdot Q^T)$. It is again efficiently solved by sorting the values of vector $\sum_{P\neq Q\in\Xi} R_t^{PQ}$.

3 Validating the *c-SKL* method: The "siblings" experiment

To validate the proposed dataset similarity method, we randomly divided each dataset into two mutually exclusive parts, A and B (subsequently referred as "siblings") and compute SKL among all pairwise combinations of datasets. We then count how many times each sibling ranks first, second, etc. or greater than 10 for all combinations. We repeat random splitting 10 times and ensure that each split includes at least 20 samples.

The pseudocode of the "siblings" experiment is given in Supplementary Figure 4 and the results for each technology in Supplementary Figure 5.

```
For Π = all datasets of each technology
{ Histogram[Π, 1..10] ← 0 // for rank 1,2,3 .. 10+
   For D in Π, with n≥40 samples
    { For count = 1 to 10
     { P ← random samples of size max(20,n/4) to n/2 out of D
        Q ← rest samples in D
        dPQ ← SKL(P,Q)
        rank(P) ← 1, rank(Q) ← 1
        For D* in Π, D* ≠ D
        { If SKL(D*,P) < dPQ then rank(Q) ← min( rank(Q)+1, 10)
          If SKL(D*,Q) < dPQ then rank(P) ← min( rank(P)+1, 10)}
        Increase Histogram[Π,rank(P)] by +1
        Increase Histogram[Π,rank(Q)] by +1
    } }
return( Histogram[Π,1..10] )
}
```

Supplementary Figure 4: Pseudocode of the "siblings" experiment.

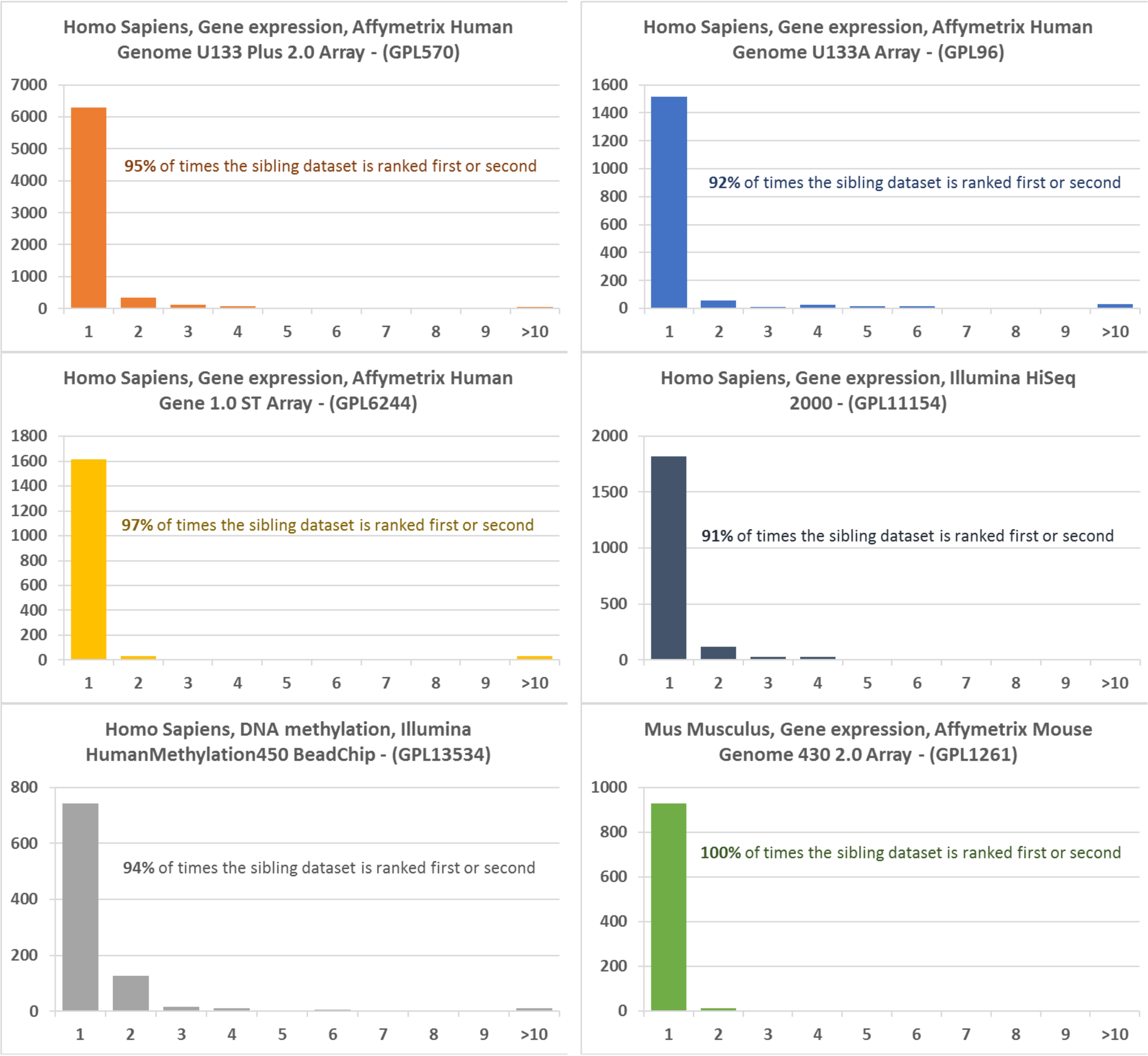

Supplementary Figure 5: Frequencies of the rank of sibling datasets for each measurement technology. On average, for a given dataset D, 95% of times its sibling dataset is ranked first or second in terms of SKL with D.

4 Validating the method that explains the SKL method based on enriched pathways

To validate the heuristic algorithm for explaining *c-SKL*, we computed the *c-SKL* achieved by varying the number of *k* probe sets (features) obtained by the explain *c-SKL* method (see section 2.6) and plotted against the *c-SKL* obtained by randomly selecting the exact same number of probe sets. We repeat this for three different pairs of similarities for each platform: the pair with the lowest, the closest to the average, and the highest similarity. In Supplementary Figure 6 we show with red dots the *c-SKL* as computed by considering only the *k* probe sets that explain the initial *c-SKL* and with grey dots the *c-SKL* of the randomly selected probe sets. Some discontinuities in red dots are noticed between successive values of *k* indicating the heuristic nature of the procedure.

Pair of datasets with the ***lowest*** *c-SKL*	Pair of datasets with an ***average*** *c-SKL*	Pair of datasets with the ***highest*** *c-SKL*

Homo Sapiens, Gene expression, Affymetrix Human Genome U133 Plus 2.0 Array - (**GPL570**)

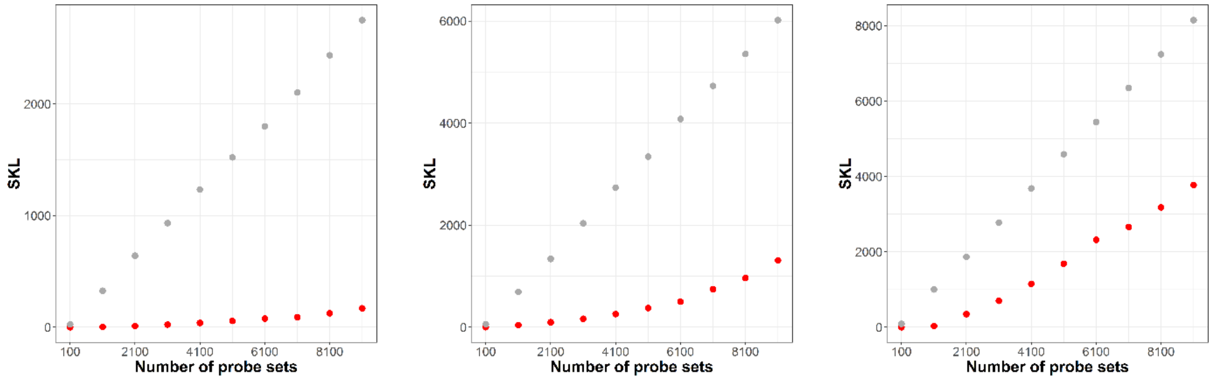

Homo Sapiens, Gene expression, Affymetrix Human Genome U133A Array - (**GPL96**)

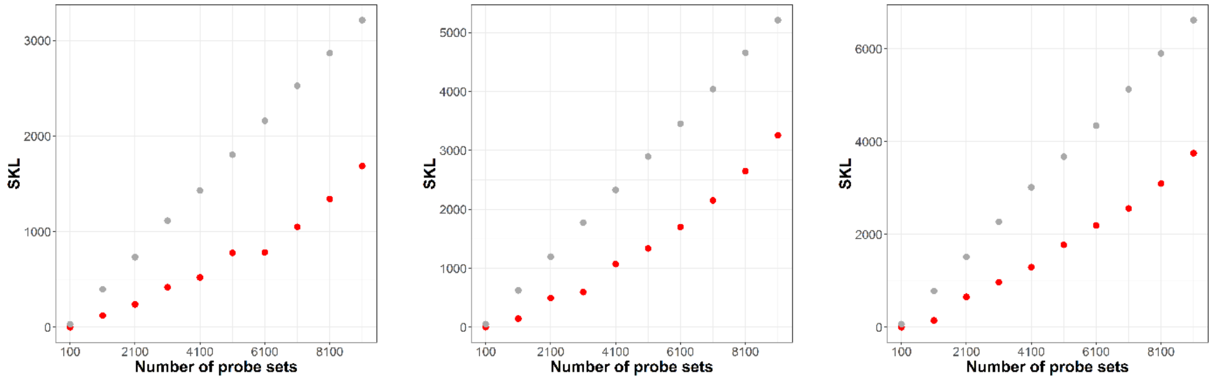

Homo Sapiens, Gene expression, Affymetrix Human Gene 1.0 ST Array - (**GPL6244**)

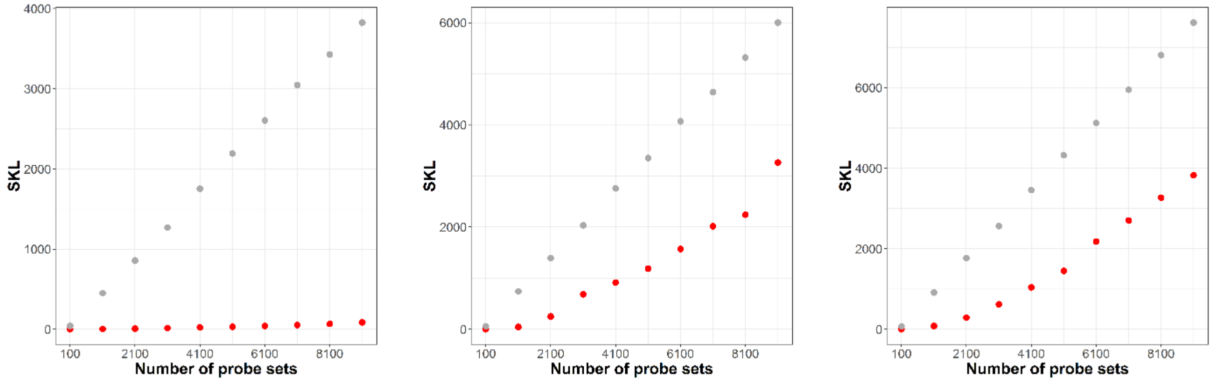

Homo Sapiens, Gene expression, Illumina HiSeq 2000 – (**GPL11154**)

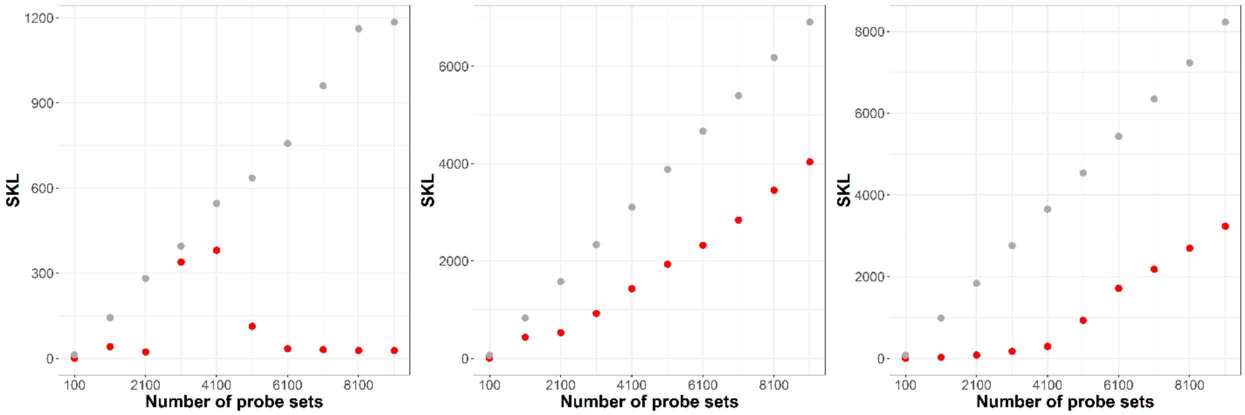

Homo Sapiens, DNA methylation, Illumina HumanMethylation450 BeadChip - (**GPL13534**)

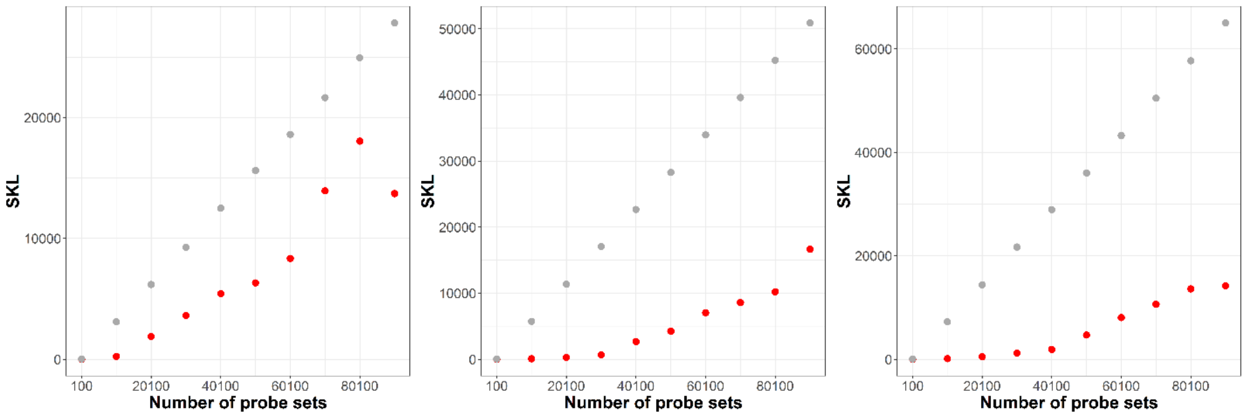

Mus Musculus, Gene expression, Affymetrix Mouse Genome 430 2.0 Array - (**GPL1261**)

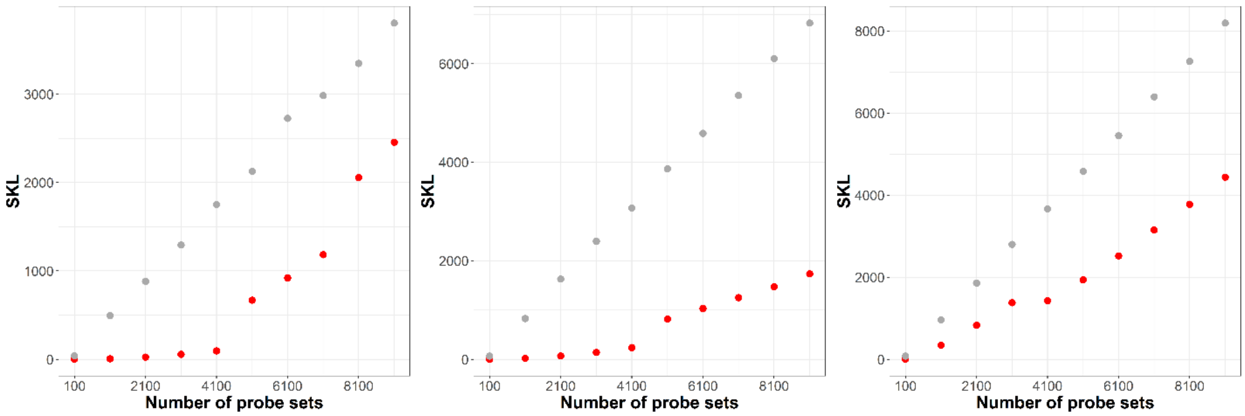

Supplementary Figure 6: Validating the explain c-SKL heuristic method. Red dots: c-SKL versus the number of top k probe sets that best explain the similarity of two datasets for three different pairs of datasets measured by each of the six measurement platforms. Grey dots: c-SKL computed using the same number of randomly selected probe sets.

5 Visualizing the Landscape of -omics data.

The *c-SKL* between every pair of datasets is computed for each of the six technologies. The results can be visualized as networks where each dataset is represented by a node and each edge between datasets X and Y denotes an *c-SKL* that is statistically significant as described in Section 2.4. In Supplementary Figure 7 we show bar plots of the number of statistically significant connections of each dataset (a.k.a. node degree in network science, or neighbors) for each measurement platform. It is interesting to note that the percentage of "isolated" datasets, namely datasets that are not statistically significantly similar to any other dataset measured by the same platform, differs among platforms. Most isolated datasets have been measured by GPL11154, an RNASeq platform and GPL13534, a DNA methylation microarray platform. Moreover, few datasets in each platform appear to have a relatively high number of connections to other datasets.

Subsequently, we provide four graphs from each technology at different thresholds regarding the number of statistically significance edges (E=10, 20, 80 and all edges). These graphs can be further explored using network analysis methods to either discover expected biological knowledge (i.e. exploring cliques or communities of diseases), or more interestingly, to discover novel biological knowledge hidden in the molecular underpinnings of omics measurements.

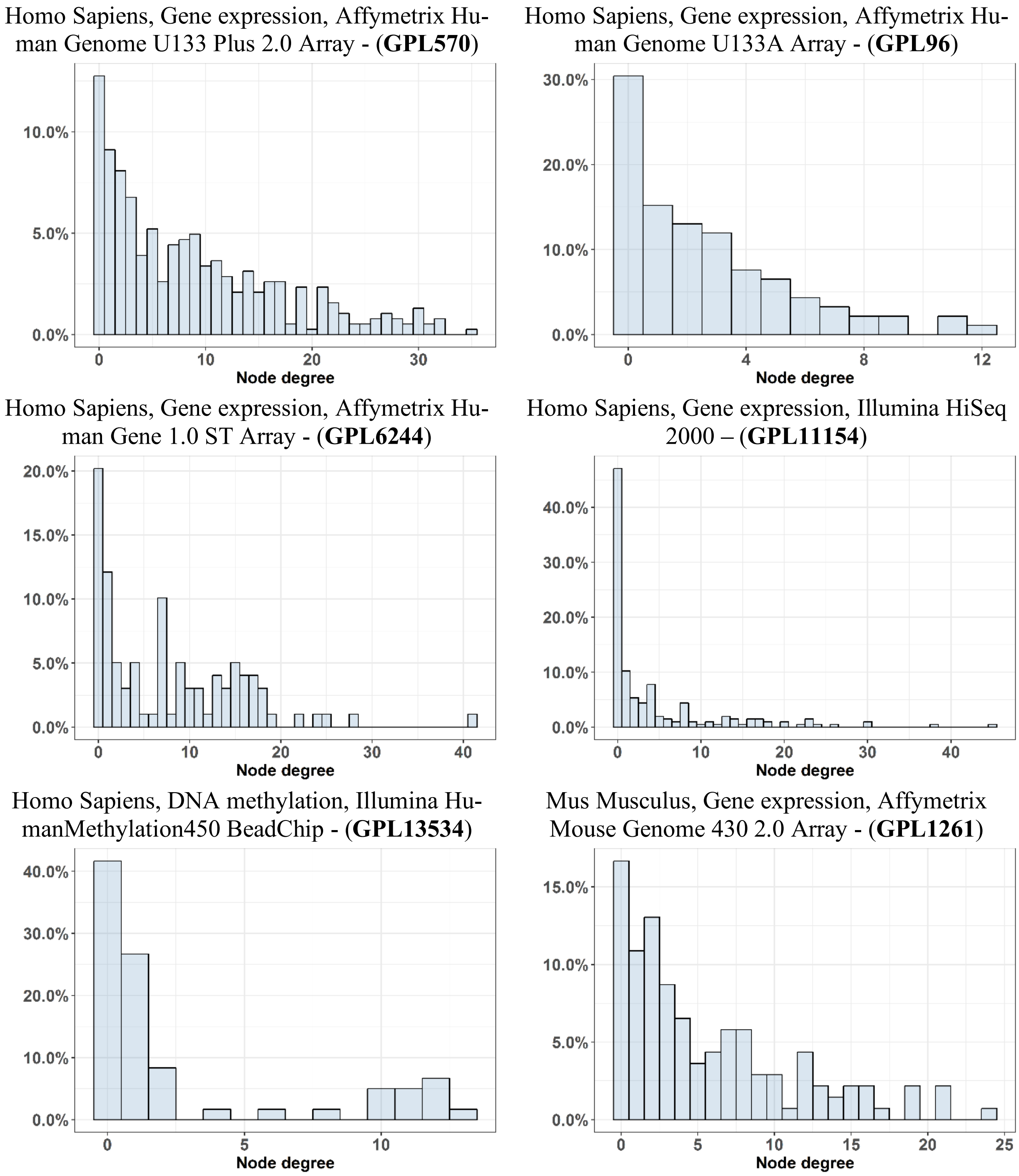

Supplementary Figure 7: Bar plot of the number of statistically significant connections of each dataset (a.k.a. node degree in network science) for each measurement technology.

Homo Sapiens, Gene expression, Affymetrix Human Genome U133 Plus 2.0 Array - (**GPL570**)

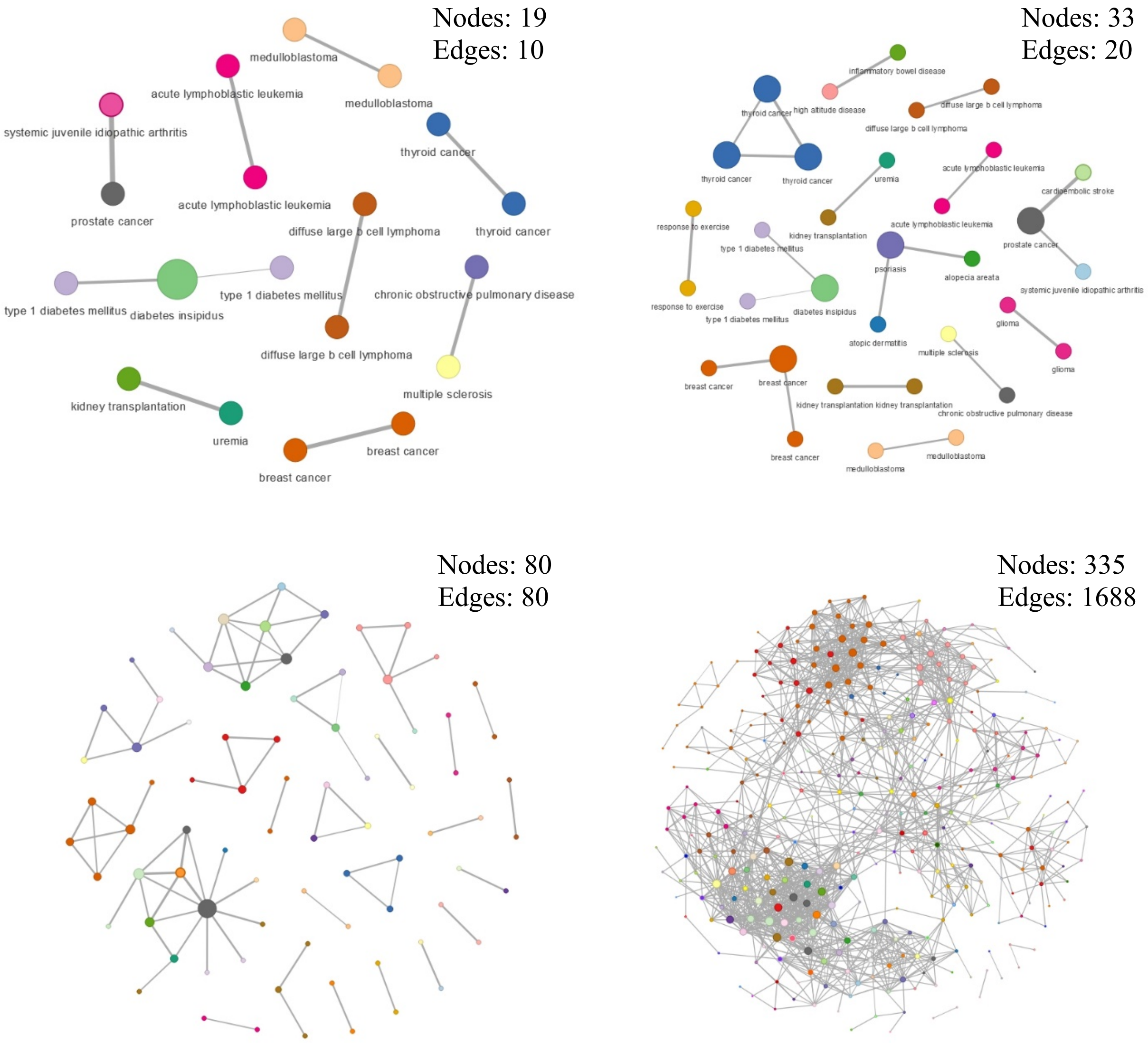

Homo Sapiens, Gene expression, Affymetrix Human Genome U133A Array - (**GPL96**)

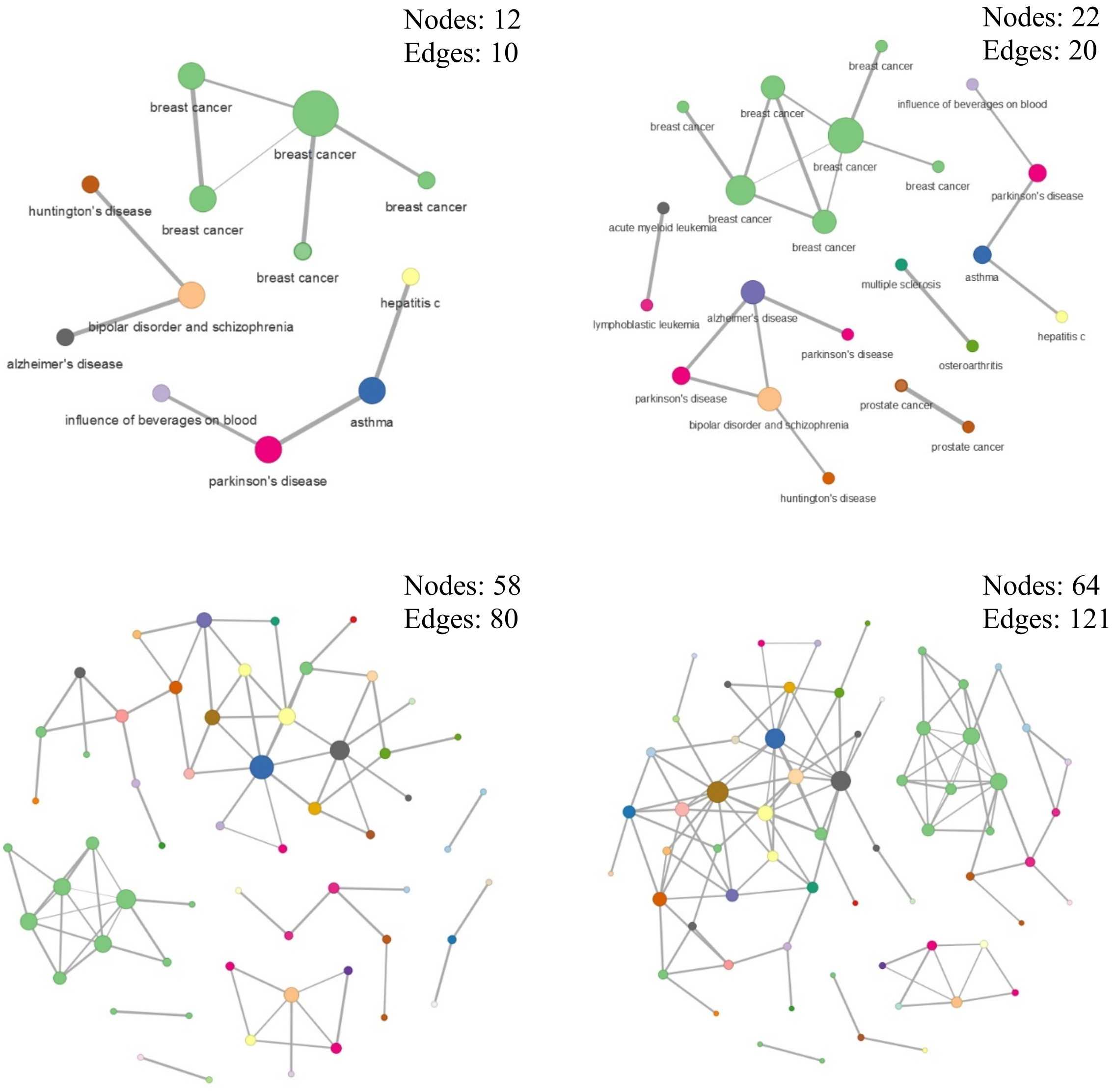

Homo Sapiens, Gene expression, Affymetrix Human Gene 1.0 ST Array - (**GPL6244**)

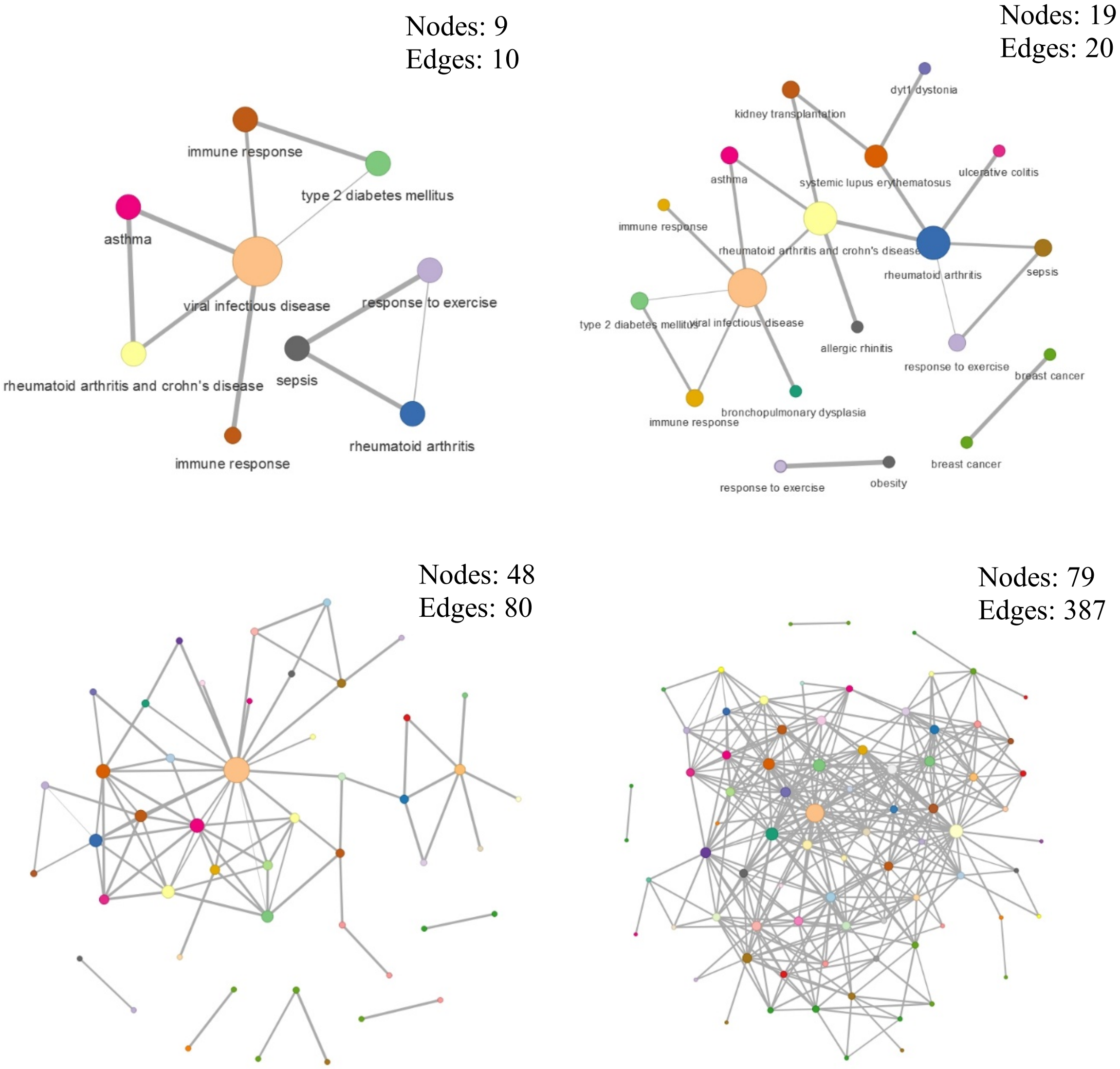

Homo Sapiens, Gene expression, Illumina HiSeq 2000 – (**GPL11154)**

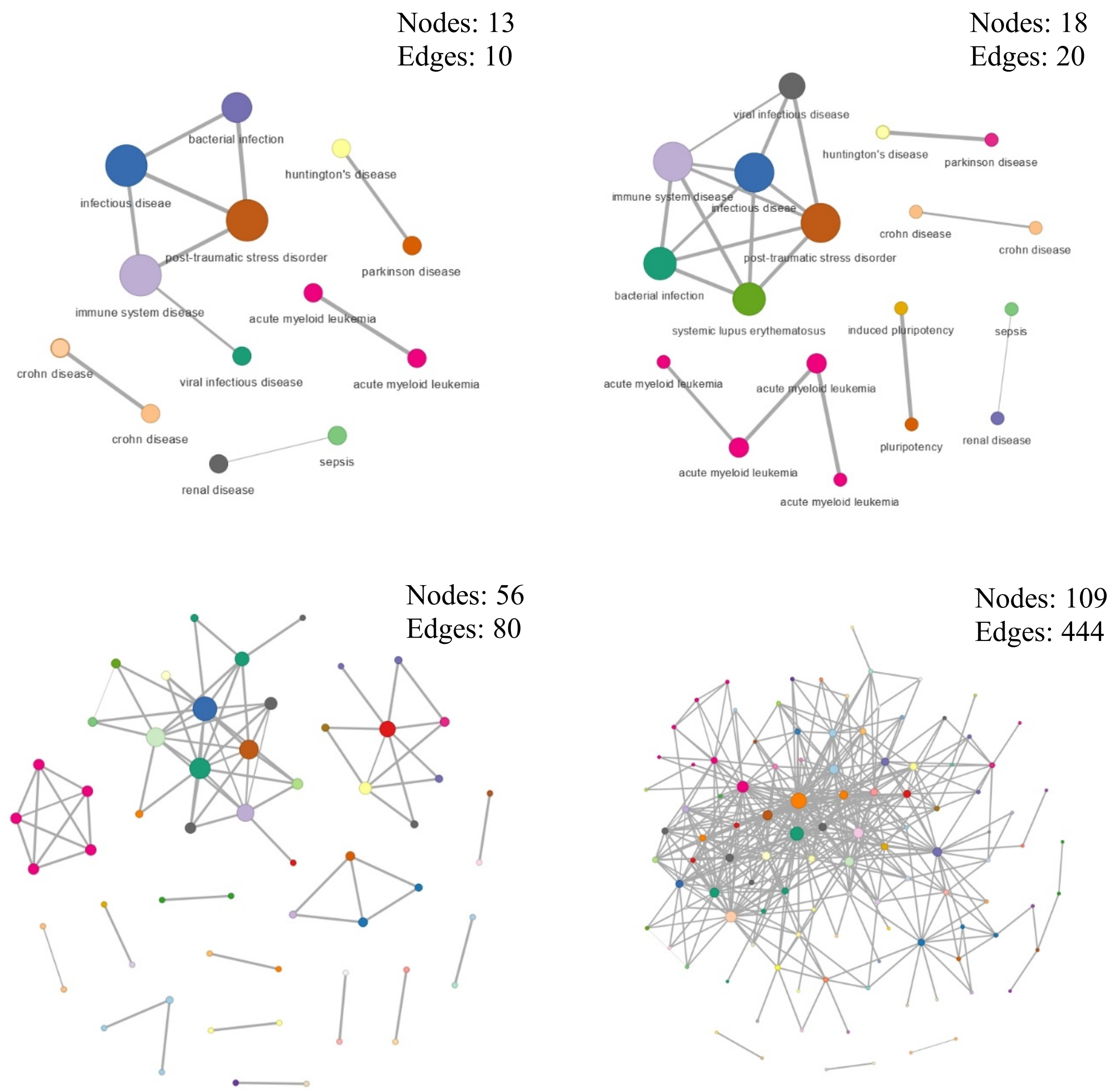

Homo Sapiens, DNA methylation, Illumina HumanMethylation450 BeadChip - (**GPL13534**)

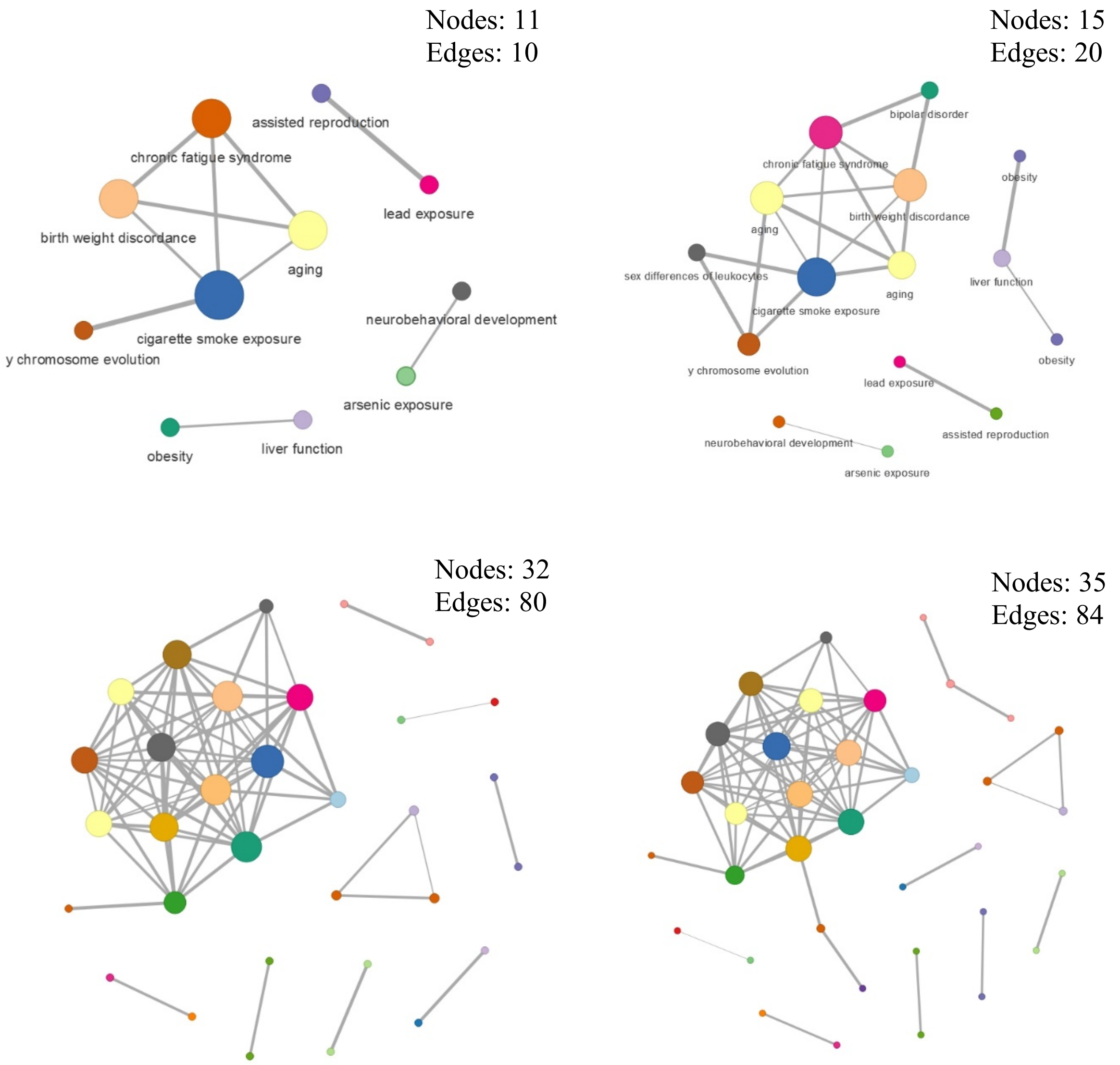

Mus Musculus, Gene expression, Affymetrix Mouse Genome 430 2.0 Array - (**GPL1261**)

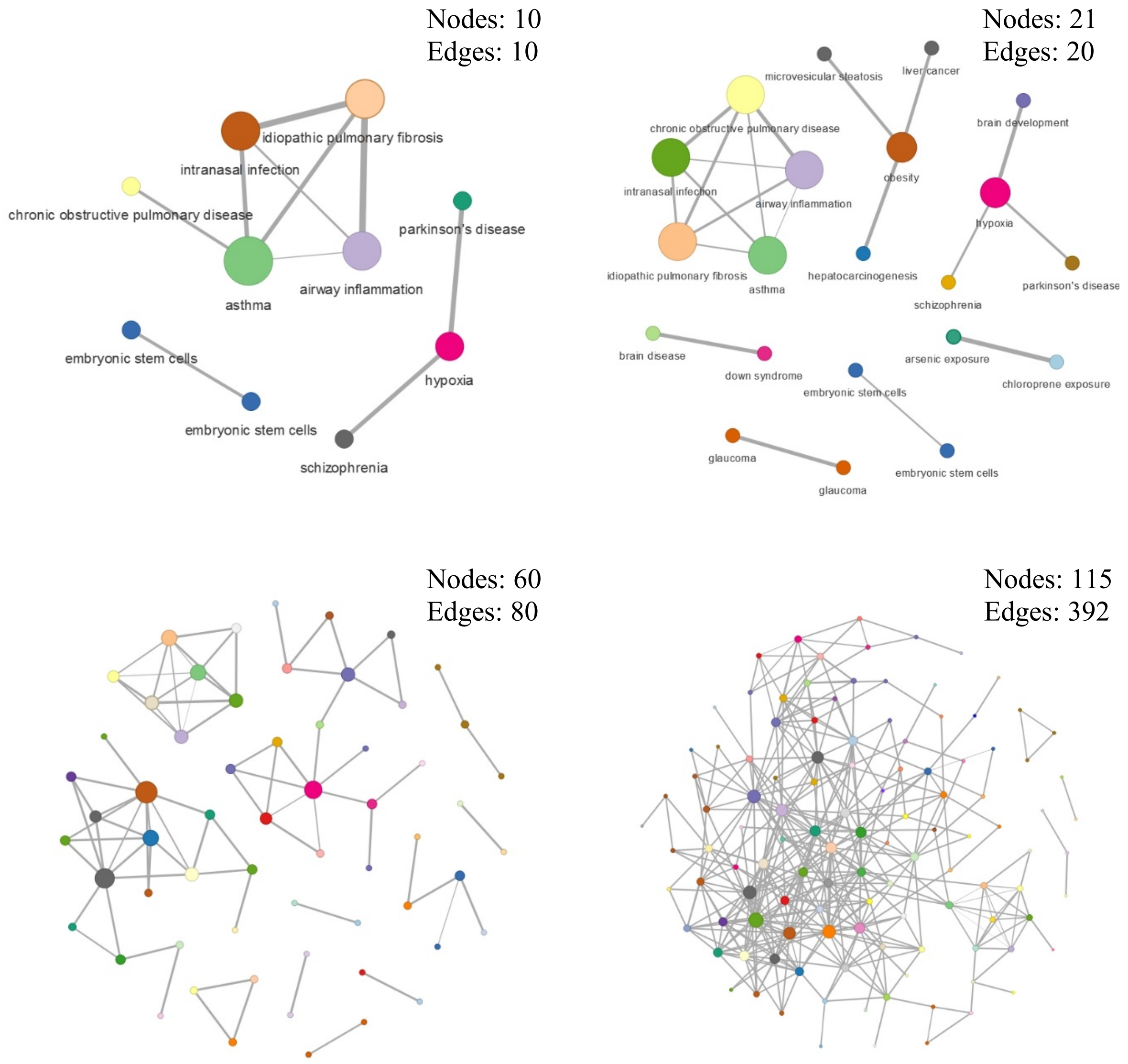

6 From dataset similarity network to disease similarity network.

Omics datasets may be found similar for several reasons (tissue, disease, experimental design, e.t.c.). Since most of the textual metadata in relevant databases is not provided in a standardized format for larger scale analysis, rather it is often optionally provided by the researcher in an unstructured way, we are restricted to massively explore the *c-SKL* similarity only on the basis of the disease or phenotype that each dataset is referred to, as provided in BioDataome and verified by experts. By substituting datasets ids with their respective disease/ phenotype, we can explore disease to disease similarities and systematically discover interesting relations. For example, when we remove all pairs of identical diseases (e.g. breast cancer-breast cancer), which account for 18,6% of all pairs (2727 in total) measuring *Homo Sapiens* samples, we can count how many times different diseases connect. In Supplementary Figure 8 we show the number of identical pairs for every disease or phenotype. In Supplementary Figure 9 we show disease to disease networks as these are formed from non-identical dataset pairs (e.g. breast cancer-lung cancer). Edge weight represents the frequency of each pair. For example, a weight of 34 in the lung cancer-breast cancer edge means that we have found 34 different pairs of datasets connecting lung cancer with breast cancer. We visualized the network in three different minimum edge weights E_w:{ >10, >5, >3 }.

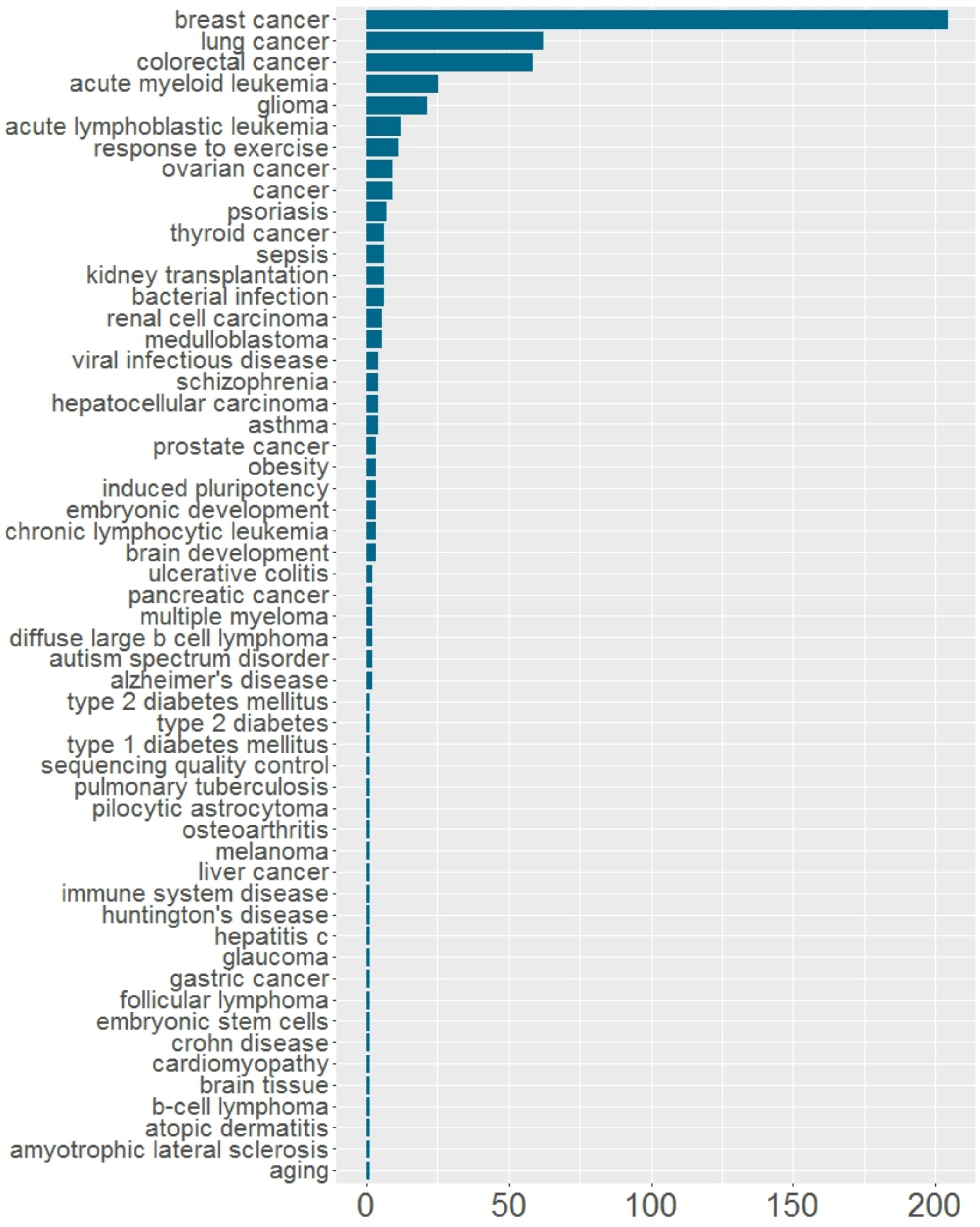

Supplementary Figure 8: Frequency of identical phenotype to phenotype similarities. For example, a breast cancer-breast cancer connection is found in 204 different dataset pairs.

Nodes: 10, Edges: 7, min edge weight: 11

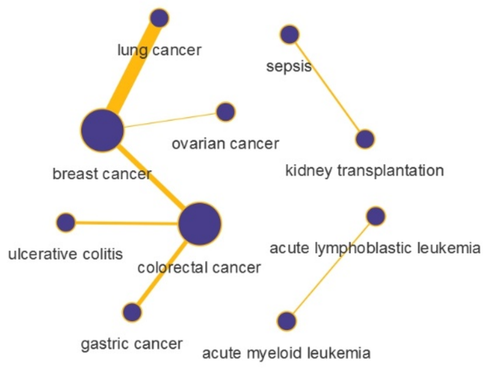

Nodes: 32
Edges: 29
min edge weight: 6

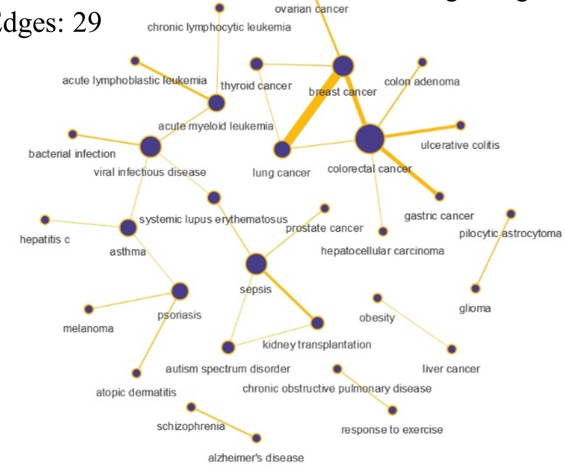

Nodes: 90
Edges: 167
min edge weight: 3

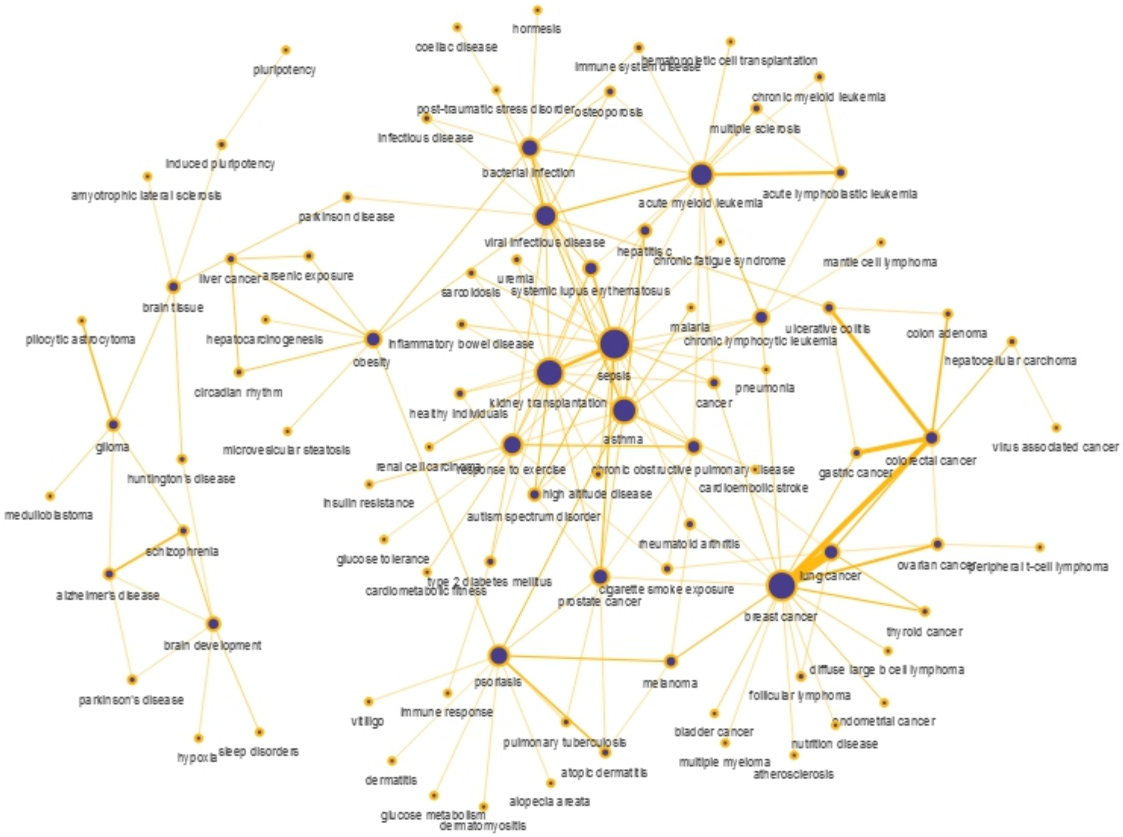

Supplementary Figure 9: Disease to disease network. Nodes represent diseases or study objects (i.e. response to exercise) and edge weight represents the frequency of the disease-disease relation. Node size increases with the number of incoming edges.

Disease/ study object	Disease/ study object	Frequency
**Breast Cancer**	Lung Cancer	34
**Breast Cancer**	Colorectal Cancer	20
**Colorectal Cancer**	Gastric Cancer	18
**Colorectal Cancer**	Ulcerative Colitis	15
**Kidney Transplantation**	Sepsis	13
**Acute Lymphoblastic Leukemia**	Acute Myeloid Leukemia	12
**Breast Cancer**	Ovarian Cancer	11
**Bacterial Infection**	Viral Infectious Disease	9
**Colon Adenoma**	Colorectal Cancer	9
**Atopic Dermatitis**	Psoriasis	8
**Alzheimer's Disease**	Schizophrenia	8
**Glioma**	Pilocytic Astrocytoma	8
**Acute Myeloid Leukemia**	Viral Infectious Disease	7
**Breast Cancer**	Thyroid Cancer	7
**Chronic Obstructive Pulmonary Disease**	Response to Exercise	7
**Colorectal Cancer**	Lung Cancer	7
**Melanoma**	Psoriasis	7
**Prostate Cancer**	Sepsis	7
**Sepsis**	Systemic Lupus Erythematosus	7
**Acute Myeloid Leukemia**	Chronic Lymphocytic Leukemia	6
**Asthma**	Hepatitis C	6
**Asthma**	Psoriasis	6
**Asthma**	Viral Infectious Disease	6
**Autism Spectrum Disorder**	Kidney Transplantation	6
**Autism Spectrum Disorder**	Sepsis	6
**Colorectal Cancer**	Hepatocellular Carcinoma	6
**Liver Cancer**	Obesity	6
**Lung Cancer**	Thyroid Cancer	6
**Systemic Lupus Erythematosus**	Viral Infectious Disease	6
**Acute Myeloid Leukemia**	Multiple Sclerosis	5
**Asthma**	Chronic Lymphocytic Leukemia	5
**Asthma**	Chronic Obstructive Pulmonary Disease	5
**Asthma**	Kidney Transplantation	5
**Asthma**	Malaria	5
**Bacterial Infection**	Obesity	5
**Brain Development**	Schizophrenia	5
**Breast Cancer**	Endometrial Cancer	5
**Breast Cancer**	Gastric Cancer	5
**Breast Cancer**	Melanoma	5
**Circadian Rhythm**	Liver Cancer	5

**Circadian Rhythm**	Obesity	5
**Kidney Transplantation**	Renal Cell Carcinoma	5
**Acute Myeloid Leukemia**	Bacterial Infection	4
**Acute Myeloid Leukemia**	Hematopoietic Cell Transplantationβ	4
**Asthma**	Autism Spectrum Disorder	4
**Asthma**	Bacterial Infection	4
**Asthma**	Chronic Lymphocytic Leukemia	4
**Asthma**	Hepatitis C	4
**Asthma**	Malaria	4
**Asthma**	Prostate Cancer	4
**Bacterial Infection**	Obesity	4
**Bacterial Infection**	Osteoporosis	4
**Bacterial Infection**	Systemic Lupus Erythematosus	4
**Brain Tissue**	Glioma	4
**Brain Tissue**	Huntington's Disease	4
**Brain Tissue**	Parkinson Disease	4
**Breast Cancer**	Cancer	4
**Breast Cancer**	Diffuse Large B Cell Lymphoma	4
**Breast Cancer**	Gastric Cancer	4
**Cancer**	Sepsis	4
**Cardiometabolic Fitness**	Response to Exercise	4
**Glioma**	Medulloblastoma	4
**Glioma**	Schizophrenia	4
**Hepatitis C**	Sepsis	4
**High Altitude Disease**	Sepsis	4
**Inflammatory Bowel Disease**	Sepsis	4
**Insulin Resistance**	Response to Exercise	4
**Kidney Transplantation**	Prostate Cancer	4
**Kidney Transplantation**	Response to Exercise	4
**Kidney Transplantation**	Systemic Lupus Erythematosus	4
**Kidney Transplantation**	Viral Infectious Disease	4
**Obesity**	Psoriasis	4
**Obesity**	Viral Infectious Disease	4
**Osteoporosis**	Viral Infectious Disease	4
**Post-Traumatic Stress Disorder**	Viral Infectious Disease	4
**Prostate Cancer**	Psoriasis	4
**Psoriasis**	Pulmonary Tuberculosis	4
**Response to Exercise**	Sepsis	4
**Response to Exercise**	Systemic Lupus Erythematosus	4

**Response to Exercise**	Type 2 Diabetes Mellitus	4
**Response to Exercise**	Viral Infectious Disease	4
**Sarcoidosis**	Sepsis	4
**Sepsis**	Viral Infectious Disease	4
**Ulcerative Colitis**	Viral Infectious Disease	4

Supplementary Table 2: Disease to disease similarities found in Homo Sapiens gene expression datasets. Frequency denotes the number of times a similarity is found in different dataset pairs.

7 Molecular underpinnings of breast and lung cancer

As already mentioned, our proposed dataset similarity measure, captures and compares the overall "idiosyncrasy" of a dataset. Two or more datasets may look similar for several reasons (e.g. experimental conditions, population characteristics, phenotypic characteristics etc.). Therefore, the most influential features, as resulted by the explain *c-SKL* method, encode the ensemble of these reasons and it is extremely difficult to isolate a single similarity cause.

To further study disease as the prevalent reason of similarity, we focus on two cancer diseases, breast and lung cancer, and try to infer how biological mechanisms are depicted on functional enrichment analysis.

First, we discover the largest breast cancer clique consisting of 10 datasets, measured with GPL570. A clique in graph theory terms is a subset of vertices of an undirected graph such that every two distinct vertices in the clique are adjacent. Next, we select the first 1000 probe sets that simultaneously explain clique similarity (see 2.5 for details on explaining *c-SKL*), map them to their corresponding gene symbols and perform enrichment analysis.

The 10 datasets are: "GSE26639" (340 breast cancer tissue samples, 220 HER2 negative and 120 HER2 positive), "GSE20685" (327 breast cancer tissue samples, 252 HER2 negative, 75 HER2 positive), "GSE43358" (57 breast cancer tissue samples, 14 HER2 positive, 33 HER2 negative), "GSE58984" (94 HER2 positive breast cancer tissue samples), "GSE43365"(155 breast cancer tissue samples), "GSE48905"(65 breast cancer tissue samples, ER or PGR positive), "GSE9195"(255 breast cancer tissue samples, ER or PGR positive), "GSE48906"(96 breast cancer tissue samples), "GSE6532"(666 breast cancer tissue samples, ER positive), "GSE16391"(48 breast cancer tissue samples all ER or PGR positive, of them 8 HER2 positive).

Enrichment analysis is a computational method for inferring knowledge about an input gene set by comparing it to annotated gene sets representing prior biological knowledge [6]. We performed enrichment analysis with the ReactomePA, an R/Bioconductor package [7] and clusterProfiler, an R/Bioconductor package [8]. clusterProfiler implements methods to analyze and visualize functional profiles of genomic coordinates, gene and gene clusters and supports several ontologies/pathways, like Gene Ontology Molecular Signatures Database [9]. Overall, we performed enrichment analysis on Reactome pathways, KEGG pathways and Hallmark gene sets [10]. We found 55, 100 and 9 significantly enriched (qvalue< 0.05) pathways in KEGG, Reactome and Hallmark databases, out of 315, 1362 and 50, respectively. In Supplementary Figure 10 we show the results of Gene Ontology analysis and in Supplementary Supplementary Table 3 the 20 most statistically significantly enriched pathways, for the genes that explain the breast cancer clique.

A general notice in Supplementary Supplementary Table 3 is that the gene list that explains the breast cancer clique consists of genes found in all cellular compartments (nucleus, cytoplasm, organelles) and it also enriches a good homology of specific pathways in all pathway databases. Moreover, several immune related genes that enrich either immune pathways, or pathways related to infiltration with immune cells, or viral infection, appear in almost all pathways shown in Supplementary Supplementary Table 3 (see orange shaded cells). This finding is not surprising, since immune infiltrate related to breast cancer evolution has been already reported [11–14] along with the expression of immune signatures in breast cancer cells [15,16] and the activation of immune pathways in breast cancer through steroid signaling [17]. Likewise, inflammation-related genes, that support the interaction of inflammation and cancer hypothesis, have been also studied [18–21].

Actin cytoskeleton dynamics related genes (see grey shaded cells in Supplementary Supplementary Table 3 for related pathways), directly related to tumor cell motility and metastasis [22–25] and interaction of cancer cells with the extracellular matrix and stromal cells [26–31] are currently explored as novel therapeutic targets in breast cancer.
However, it is surprising that there are no cell cycle, mitosis, DNA repair, p53, ERBB or steroid hormone receptor pathways among the top 20. This indicating that in a massive analysis like this, what is most similar among the breast cancer datasets, even comprised of all the subtypes known (ER and PGR positive, HER2 positive and triple negative), obviously are the immunological pathways. Interestingly, breast cancer in general is the least responsive to checkpoint immunotherapy, except the triple-negative subtype [32]. However, these profiles can probably be helpful in finding the reasons for immunotherapy resistance.

Pathways/ molecular databases	qvalue
Reactome Pathways	
Immunoregulatory interactions between a Lymphoid and a non-Lymphoid cell	3,14E-26
Signaling by Interleukins	2,74E-16
Phosphorylation of CD3 and TCR zeta chains	2,71E-14
Interleukin-2 family signaling	3,20E-14
Generation of second messenger molecules	4,01E-14
Translocation of ZAP-70 to Immunological synapse	4,01E-14
Costimulation by the CD28 family	9,36E-14
PD-1 signaling	1,13E-12
TCR signaling	1,31E-12
Interferon gamma signaling	2,59E-11
GPVI-mediated activation cascade	7,53E-10
Antigen activates B Cell Receptor (BCR) leading to generation of second messengers	1,57E-09
Chemokine receptors bind chemokines	4,16E-09
Interleukin-3, 5 and GM-CSF signaling	4,16E-09
Signaling by the B Cell Receptor (BCR)	1,63E-08
DAP12 signaling	2,26E-08
Cell surface interactions at the vascular wall	4,22E-08
Interleukin-10 signaling	5,49E-08
FCERI mediated Ca+2 mobilization	6,19E-08
Downstream TCR signaling	1,96E-07
KEGG Pathways	
Th1 and Th2 cell differentiation	4,06E-20
Primary immunodeficiency	2,16E-19
Allograft rejection	9,22E-18
Hematopoietic cell lineage	9,22E-18
Natural killer cell mediated cytotoxicity	9,22E-18
Th17 cell differentiation	1,31E-17
Cytokine-cytokine receptor interaction	5,75E-17
Intestinal immune network for IgA production	1,10E-16
Cell adhesion molecules (CAMs)	1,18E-16
Chemokine signaling pathway	1,21E-14
Graft-versus-host disease	1,21E-14
T cell receptor signaling pathway	1,67E-14
Type I diabetes mellitus	2,90E-14
Inflammatory bowel disease (IBD)	5,76E-14
Leishmaniasis	8,01E-14
Viral myocarditis	8,01E-14
Autoimmune thyroid disease	1,11E-13
Antigen processing and presentation	1,78E-13
NF-kappa B signaling pathway	2,20E-13
B cell receptor signaling pathway	3,48E-12
Hallmark gene sets- Molecular Signatures Database	

HALLMARK_ALLOGRAFT_REJECTION	3,27E-61
HALLMARK_INTERFERON_GAMMA_RESPONSE	1,06E-26
HALLMARK_INFLAMMATORY_RESPONSE	3,44E-13
HALLMARK_IL6_JAK_STAT3_SIGNALING	5,22E-09
HALLMARK_COMPLEMENT	1,29E-07
HALLMARK_IL2_STAT5_SIGNALING	1,29E-07
HALLMARK_KRAS_SIGNALING_UP	1,29E-07
HALLMARK_INTERFERON_ALPHA_RESPONSE	1,50E-05
HALLMARK_TNFA_SIGNALING_VIA_NFKB	0,00038

	Immune related including viral and inflammation
	Major Signaling Pathways
	Focal adhesion and ECM, including actin

Supplementary Table 3: Top 20 most significantly enriched pathways that explain the breast cancer clique. Reactome and KEGG biological pathways and Hallmark gene sets are used for the enrichment analysis of the breast cancer clique top explaining genes.Statistical significance is determined by qvalue.

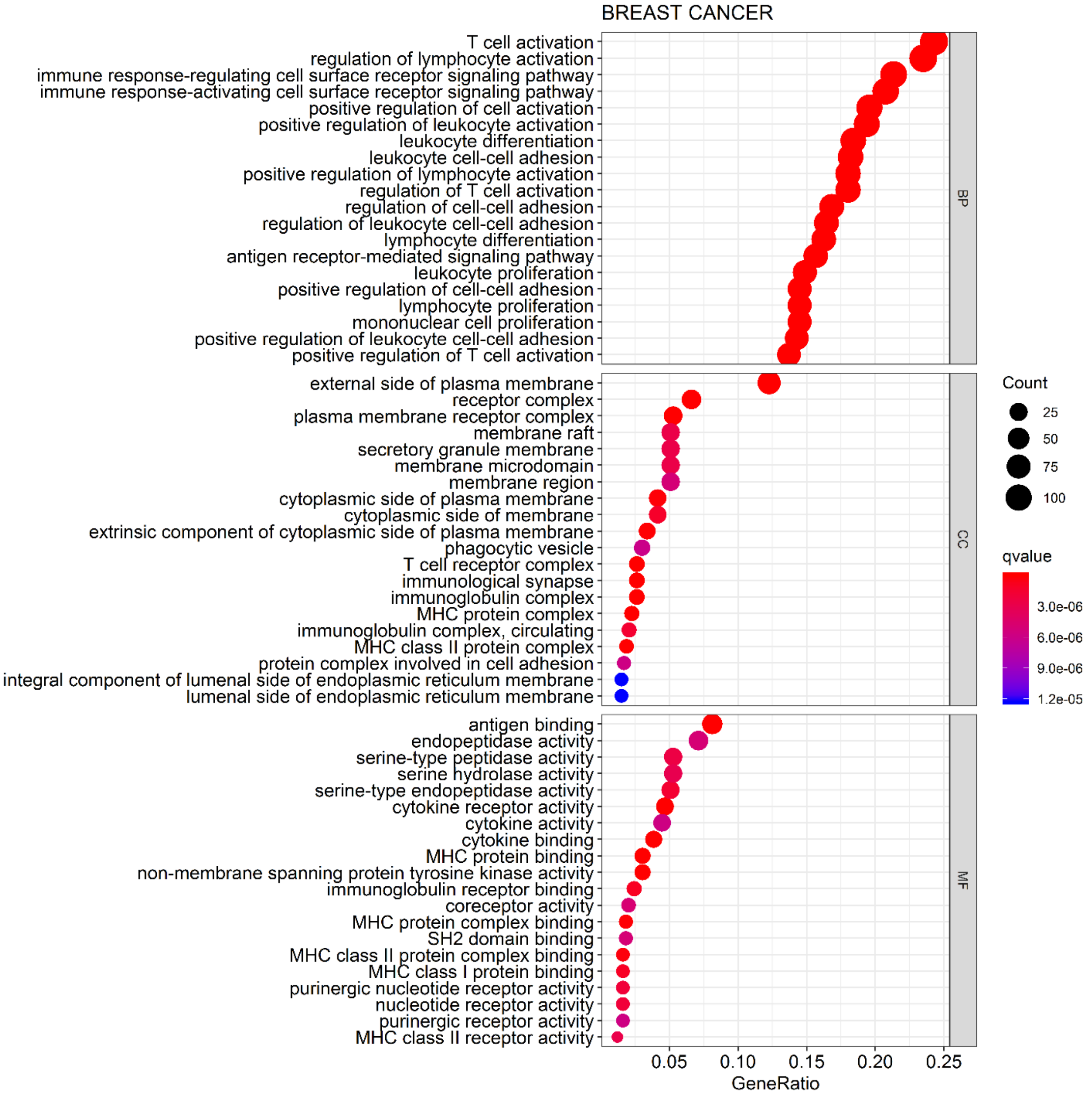

Supplementary Figure 10: Dotplot of the results of Gene Ontology overrepresentation analysis of breast cancer gene sets. BP refers to Biological Process, CC to cellular component and MF to molecular function.

The lung cancer clique consisted of nine datasets with 62, 116 and 9 significantly enriched pathways in KEGG, REACTOME and Hallmark respectively (qvalue< 0.05). Datasets in this clique included: "GSE40791"(100 normal lung tissue samples, 94 adenocarcinomas), "GSE30219" (293 lung cancer tissue samples, 85 adenocarcinoma, 37 non-tumor lung tissue), "GSE12667"(75 lung adenocarcinoma tissue samples), "GSE50081" (181 non-small cell lung cancer tissue samples), "GSE43580"(150 non-small cell lung cancer tissue samples), "GSE18842"(46 non-small cell lung

cancer tissue samples, 45 control paired lung except three), "GSE31210" (226 lung adenocarcinoma tissue samples,127 with EGFR mutation, 20 with KRAS mutation, 11 with EML4-ALK fusion and 68 wild type), "GSE10245" (40 adenocarcinoma, 18 squamous cell carcinoma), "GSE33532"(80 non-small cell lung cancer tissue samples and 20 normal lung samples).

Consistently, the majority of pathways were related to the immune system and the top-ranked in both KEGG and Hallmark was the Allograft Rejection pathway (Supplementary Supplementary Table 4). Interestingly, there were several shared genes between the three databases (Reactome, KEGG and Hallmark), and when inserting the gene lists of KEGG and Hallmark in the KEGG Allograft Rejection pathway, we find most of the genes of the pathway cross-correlated (Supplementary Figure 11). The Allograft Rejection pathway was built upon genes discovered to be deregulated in rejected transplant kidneys. Interestingly this comes up as the top pathway in lung cancer, but also in breast cancer Supplementary Supplementary Table 3. The current knowledge of how lung cancer escapes immunosurveillance by deregulating several checkpoints as *PD1* and *CTLA4* is recapitulated through the key genes *CD28*, *CD40*, *CD40* ligand, *CD80* and *CD86* [33–35].

Perforin (*PRF1*) is a gene that encodes a protein released from cytotoxic T-lymphocytes that forms membrane pores that allow the release of granzymes and subsequent cytolysis of target cells. Mutations in this gene can cause familial hemophagocytic lymphohistiocytosis type 2 (FHL2), a rare and lethal autosomal recessive disorder of early childhood [36]. Finally, several histocompatibility complex genes in this pathway were correlated, including major histocompatibility complex, class II, DR beta 1 (*HLA-DRB1*), associated to asthma, lupus, thyroiditis, diabetes type 1 and rheumatoid arthritis, all well-known side-effects of immunotherapy currently used for lung cancer. Using the current analytical method in many datasets revealed novel features in lung cancer that has been obscure in previous microarray studies and confers potential therapeutic consequences.

Pathways/ molecular databases	qvalue
**Reactome Pathways**	
Immunoregulatory interactions between a Lymphoid and a non-Lymphoid cell	1,87E-39
Signaling by Interleukins	6,55E-18
Neutrophil degranulation	1,09E-16
Interferon gamma signaling	4,09E-16
Generation of second messenger molecules	1,43E-15
Interleukin-2 family signaling	1,99E-15
Translocation of ZAP-70 to Immunological synapse	8,60E-15
PD-1 signaling	1,06E-14
Costimulation by the CD28 family	2,93E-14
Phosphorylation of CD3 and TCR zeta chains	1,49E-13
Antigen processing-Cross presentation	1,09E-11
TCR signaling	1,01E-10
Interferon Signaling	5,82E-10
Interleukin-10 signaling	6,35E-10
G-protein beta:gamma signalling	1,18E-09
G beta:gamma signalling through PI3Kgamma	4,60E-09
Interleukin-3, 5 and GM-CSF signaling	4,60E-09
GPVI-mediated activation cascade	6,25E-09
Cell surface interactions at the vascular wall	9,77E-09
Interleukin receptor SHC signaling	4,39E-08
**KEGG Pathways**	
Allograft rejection	1,67E-24
Staphylococcus aureus infection	9,20E-23
Cell adhesion molecules (CAMs)	1,67E-22
Graft-versus-host disease	1,05E-20
Leishmaniasis	4,86E-20
Autoimmune thyroid disease	5,35E-20
Hematopoietic cell lineage	2,07E-19
Phagosome	4,90E-19
Type I diabetes mellitus	6,08E-19
Natural killer cell mediated cytotoxicity	8,14E-19
Intestinal immune network for IgA production	2,34E-17
Osteoclast differentiation	2,34E-17
Tuberculosis	1,68E-16
Antigen processing and presentation	1,68E-16
Viral myocarditis	1,88E-16
Th1 and Th2 cell differentiation	2,48E-16
Cytokine-cytokine receptor interaction	8,37E-15
Rheumatoid arthritis	1,19E-14
Th17 cell differentiation	1,93E-14
Primary immunodeficiency	3,52E-14

Hallmark gene sets- Molecular Signatures Database	
HALLMARK_ALLOGRAFT_REJECTION	1,93E-44
HALLMARK_INTERFERON_GAMMA_RESPONSE	9,07E-12
HALLMARK_INFLAMMATORY_RESPONSE	3,29E-04
HALLMARK_COMPLEMENT	2,10E+04
HALLMARK_INTERFERON_ALPHA_RESPONSE	6,41E+04
HALLMARK_IL6_JAK_STAT3_SIGNALING	1,79E+05
HALLMARK_KRAS_SIGNALING_UP	4,13E+07
HALLMARK_IL2_STAT5_SIGNALING	1,24E+09
HALLMARK_TNFA_SIGNALING_VIA_NFKB	0.00169

	Immune related including viral and inflammation
	Major Signaling Pathways
	Focal adhesion and ECM, including actin

Supplementary Table 4: Top 20 most significantly enriched pathways that explain the lung cancer clique. Reactome and KEGG biological pathways and Hallmark gene sets are used for the enrichment analysis of the lung cancer clique top explaining genes.Statistical significance is determined by qvalue.

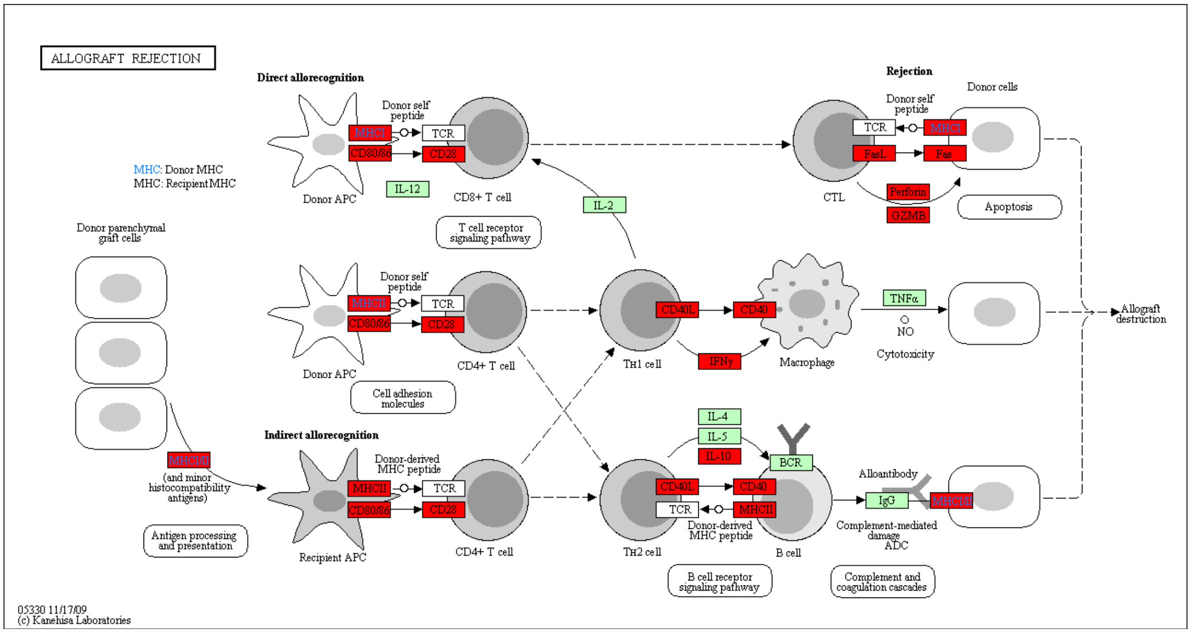

Supplementary Figure 11: KEGG Allograft Rejection pathway in the lung cancer clique of 9 datasets. The inter-correlated genes in the REACTOME were highly similar producing an identical map (red boxes show genes that are inter-correlated in all 9 datasets).

8 Comparing findings from different measurement technologies

Although different measurement technologies include different probe sets, we hypothesize that similar studies should imprint similar disease mechanisms in their gene expression patterns. To study this hypothesis, we compared gene sets from two different measurement technologies (Affymetrix Human Genome U133 Plus 2.0 Array - **GPL570** and Illumina HiSeq 2000 - **GPL11154**) and two different disease cliques, Acute Myeloid Leukemia (AML) that includes five datasets from each technology and psoriasis, including three different datasets from each technology. Specifically, for each clique we vary the number of probe sets that explain the clique from 100 to 54000. We then map probe sets to gene symbols and compute the Jaccard similarity coefficient between two gene sets of the same disease clique (i.e. AML) measured by the two different technologies, GPL570 and GPL11154 (see red dots). Each time we also compute the Jaccard similarity coefficient between the same number of randomly selected probe sets (see grey dots Supplementary Figure 12). The maximum Jaccard index between GPL570 and GPL11154 is 0.62.

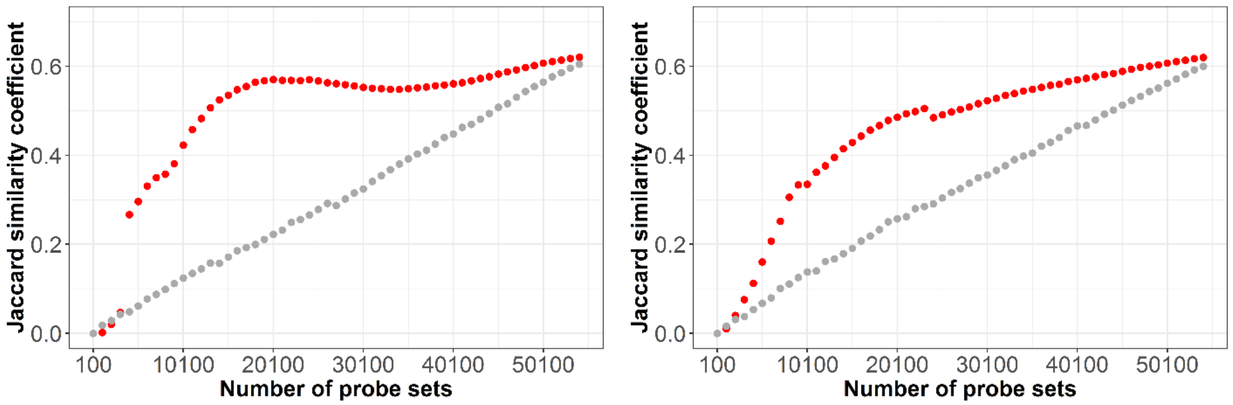

Supplementary Figure 12 Left. Jaccard similarity coefficient between two cliques of AML measured by GPL570 and GPL11154. Right. Jaccard similarity coefficient between two cliques of psoriasis measured by GPL570 and GPL11154. Red dots represent the Jaccard index of two gene sets that explain the similarity of an AML (left) and a psoriasis (right) clique of five and three different datasets respectively, for a varying number of probe sets. Grey dots represent the Jaccard index of the same number of randomly selected probe sets.

SUPPLEMENTARY REFERENCES

1. Lakiotaki, K., Vorniotakis, N., Tsagris, M., Georgakopoulos, G. & Tsamardinos, I. BioDataome: a collection of uniformly preprocessed and automatically annotated datasets for data-driven biology. *Database (Oxford).* **2018,** (2018).

2. Kibbe, W. A. *et al.* Disease Ontology 2015 update: An expanded and updated database of Human diseases for linking biomedical knowledge through disease data. *Nucleic Acids Res.* **43,** D1071–D1078 (2015).

3. Kullback, S. & Leibler, R. A. On Information and Sufficiency. *Ann. Math. Stat.* **22,** 79–86 (1951).

4. Duchi, J. in *Derivations for Linear Algebra and Optimization* (2007).

5. Floudas, C. A. & Visweswaran, V. *Quadratic Optimization*. (1995). doi:10.1007/978-1-4615-2025-2_5

6. Clark, N. & Ma'ayan, A. Introduction to Statistical methods for analyzing large data sets: gene-set enrichment analysis. *Sci. Signal.* **4,** 1–12 (2011).

7. Yu, G. & He, Q.-Y. ReactomePA: an R/Bioconductor package for reactome pathway analysis and visualization. *Mol. BioSyst.* **12,** 477–479 (2016).

8. Yu, G., Wang, L.-G., Han, Y. & He, Q.-Y. clusterProfiler: an R Package for Comparing Biological Themes Among Gene Clusters. *OMICS* **16,** 284–287 (2012).

9. Liberzon, A. *et al.* Molecular signatures database (MSigDB) 3.0. *Bioinformatics* **27,** 1739–1740 (2011).

10. Liberzon, A. *et al.* The Molecular Signatures Database Hallmark Gene Set Collection. *Cell Syst.* **1,** 417–425 (2015).

11. Pelekanou, V. *et al.* Tumor infiltrating lymphocytes and PD-L1 expression in pre- and post-treatment breast cancers in the SWOG S0800 Phase II neoadjuvant chemotherapy trial. *Mol. Cancer Ther.* (2018). doi:10.1158/1535-7163.mct-17-1005

12. Pelekanou, V. *et al.* Effect of neoadjuvant chemotherapy on tumor-infiltrating lymphocytes and PD-L1 expression in breast cancer and its clinical significance. *Breast Cancer Res.* **19,** 1–11 (2017).

13. Zhao, X. *et al.* Prognostic significance of tumor-associated macrophages in breast cancer: a meta-analysis of the literature. *Oncotarget* **8,** 30576–30586 (2015).

14. Law, A. M. K., Lim, E., Ormandy, C. J. & Gallego-Ortega, D. The innate and adaptive infiltrating immune systems as targets for breast cancer immunotherapy. *Endocr. Relat. Cancer* **24,** R123–R144 (2017).

15. Azariadis, K. *et al.* Androgen Triggers the Pro-Migratory CXCL12/CXCR4 Axis in AR-Positive Breast Cancer Cell Lines: Underlying Mechanism and Possible Implications for the Use of Aromatase Inhibitors in Breast Cancer. *Cell. Physiol. Biochem.* 66–84 (2017). doi:10.1159/000484584

16. Pelekanou, V. *et al.* Expression of TNF-superfamily members BAFF and APRIL in breast cancer: Immunohistochemical study in 52 invasive ductal breast carcinomas. *BMC Cancer* **8,** 1–9 (2008).

17. Pelekanou, V. *et al.* The estrogen receptor alpha-derived peptide ERα17p (P295-T311) exerts pro-apoptotic actions in breast cancer cells in vitro and in vivo, independently from their ERα status. *Mol. Oncol.* **5,** 36–47 (2011).

18. Guo, L. *et al.* C-reactive protein and risk of breast cancer: A systematic review and meta-

analysis. *Sci. Rep.* **5,** 1–8 (2015).

19. Wang, W., Song, X.-W. & Zhao, C.-H. Roles of programmed cell death protein 5 in inflammation and cancer (Review). *Int. J. Oncol.* **49,** 1801–1806 (2016).

20. Wei, B. *et al.* The neutrophil lymphocyte ratio is associated with breast cancer prognosis: an updated systematic review and meta-analysis. *Onco. Targets. Ther.* **9,** 5567–5575 (2016).

21. Mills, R. C. Breast Cancer Survivors, Common Markers of Inflammation, and Exercise: A Narrative Review. *Breast Cancer Basic Clin. Res.* **11,** (2017).

22. Arjonen, A., Kaukonen, R. & Ivaska, J. Filopodia and adhesion in cancer cell motility. *Cell Adhesion and Migration* (2011). doi:10.4161/cam.5.5.17723

23. Liu, S. The ROCK signaling and breast cancer metastasis. *Mol. Biol. Rep.* (2011). doi:10.1007/s11033-010-0238-4

24. O'Connor, K. & Chen, M. Dynamic functions of RhoA in tumor cell migration and invasion. *Small GTPases* **4,** 141–147 (2013).

25. Miao, Z. *et al.* Microtubule actin cross-linking factor 1, a novel potential target in cancer. *Cancer Science* (2017). doi:10.1111/cas.13344

26. Bussard, K. M., Mutkus, L., Stumpf, K., Gomez-Manzano, C. & Marini, F. C. Tumor-associated stromal cells as key contributors to the tumor microenvironment. *Breast Cancer Research* (2016). doi:10.1186/s13058-016-0740-2

27. Butti, R. *et al.* Receptor tyrosine kinases (RTKs) in breast cancer: Signaling, therapeutic implications and challenges. *Molecular Cancer* (2018). doi:10.1186/s12943-018-0797-x

28. Majidinia, M. & Yousefi, B. Breast tumor stroma: A driving force in the development of resistance to therapies. *Chem. Biol. Drug Des.* (2017). doi:10.1111/cbdd.12893

29. Roberts, K. J., Kershner, A. M. & Beachy, P. A. The Stromal Niche for Epithelial Stem Cells: A Template for Regeneration and a Brake on Malignancy. *Cancer Cell* (2017). doi:10.1016/j.ccell.2017.08.007

30. Mao, Y., Keller, E. T., Garfield, D. H., Shen, K. & Wang, J. Stromal cells in tumor microenvironment and breast cancer. *Cancer Metastasis Rev.* (2013). doi:10.1007/s10555-012-9415-3

31. Zardavas, D., Baselga, J. & Piccart, M. Emerging targeted agents in metastatic breast cancer. *Nature Reviews Clinical Oncology* (2013). doi:10.1038/nrclinonc.2013.29

32. De La Cruz, L. M. J. & Czerniecki, B. Immunotherapy for Breast Cancer is Finally at the Doorstep: Immunotherapy in Breast Cancer. *Ann. Surg. Oncol.* **25,** 2852–2857 (2018).

33. Pentcheva-Hoang, T., Chen, L., Pardoll, D. M. & Allison, J. P. Programmed death-1 concentration at the immunological synapse is determined by ligand affinity and availability. *Proc. Natl. Acad. Sci. U. S. A.* **104,** 17765–17770 (2007).

34. Kamphorst, A. O. *et al.* Rescue of exhausted CD8 T cells by PD-1-targeted therapies is CD28-dependent. *Science* **355,** 1423–1427 (2017).

35. Gettinger, S. *et al.* Impaired HLA Class I Antigen Processing and Presentation as a Mechanism of Acquired Resistance to Immune Checkpoint Inhibitors in Lung Cancer. *Cancer Discov.* **7,** 1420–1435 (2017).

36. Cetica, V. *et al.* STXBP2 mutations in children with familial haemophagocytic lymphohistiocytosis type 5. *J. Med. Genet.* **47,** 595–600 (2010).

814
